# Lethal activity of BRD4 PROTAC degrader QCA570 against bladder cancer cells

**DOI:** 10.3389/fchem.2023.1121724

**Published:** 2023-01-17

**Authors:** Qiang Wang, Baohu Li, Wenkai Zhang, Zhuoyue Li, Bo Jiang, Sichuan Hou, Shumin Ma, Chong Qin

**Affiliations:** ^1^ Department of Urology, Qingdao Municipal Hospital, Qingdao University, Qingdao, China; ^2^ Key Laboratory of Marine Drugs, Chinese Ministry of Education, School of Medicine and Pharmacy, Ocean University of China, Qingdao, China; ^3^ Laboratory for Marine Drugs and Bioproducts, Qingdao National Laboratory for Marine Science and Technology, Qingdao, China

**Keywords:** QCA570, BRD4, PROTAC, bladder cancer, targeted therapy

## Abstract

Bladder cancer is the most common malignancy of the urinary system. Efforts to identify innovative and effective therapies for bladder cancer are urgently needed. Recent studies have identified the BRD4 protein as the critical factor in regulation of cell proliferation and apoptosis in bladder cancer, and it shows promising potential for pharmacologic treatment against bladder cancer. In this study, we have evaluated the biological function of QCA570, a novel BET degrader, on multiple bladder cancer cells and explore its underlying mechanisms. QCA570 potently induces degradation of BRD4 protein at nanomolar concentrations, with a DC_50_ of ∼ 1 nM. It decreases EZH2 and c-MYC levels by transcriptional suppression and protein degradation. Moreover, the degrader significantly induces cell apoptosis and cycle arrest and shows antiproliferation activity against bladder cancer cells. These findings support the potential efficacy of QCA570 on bladder cancer.

## Introduction

Bladder cancer (BC) remains the most common malignancy of the urinary tract with an estimated 500,000 new cases and 200,000 deaths worldwide each year (Lenis et al., 2020; Dobruch and Oszczudłowski, 2021). Clinically, bladder cancer is classified as either a non-muscle-invasive bladder cancer (NMIBC), in which the muscle tissue was not affected, or a muscle-invasive bladder cancer (MIBC) (Witjes et al., 2021). MIBC has a high rate of metastasis and is more likely to lead to death (Liu et al., 2022a). Although common treatments, such as transurethral resection and systemic chemotherapy, are effective for some MIBC patients, the recurrence and distant metastasis remains at approximately 50% and the 5-year survival rate is 50%–65% (Wu et al., 2016; Xie et al., 2020). Hence, development of novel treatment strategies is necessary to improve the clinical results of treatment of patients with bladder cancer.

The bromodomain and extraterminal domain (BET) proteins including BRD2, BRD3, BRD4 and the testis-specific protein (BRDT) are known to be important epigenome readers which interact with the acetylated histones, recruit chromatin-modifying enzymes to target promoters and function as coactivators or corepressors in a context-dependent manner (Filippakopoulos et al., 2010; Yan et al., 2014; Donati et al., 2018). By regulating gene transcription both at the initiation and elongation steps, these proteins play key roles during embryogenesis and cancer development (Wang et al., 2017). As the most widely studied member, BRD4 has emerged as an attractive therapeutic target for cancer therapy (Jung et al., 2015; Duan et al., 2018). Dysregulation of BRD4 has been involved in a variety of cancers including hematological and several solid malignancies (Ozer et al., 2018; Wu et al., 2019). For example, BRD4 is significantly upregulated in melanoma tissues and treatment with BET inhibitors impairs melanoma cell proliferation and metastatic behavior (Segura et al., 2013). In acute myeloid leukemia (AML), BRD4 has been reported to be critical for disease maintenance and suppression of BRD4 led to significant antileukemic effects *in vitro* and *in vivo* (Zuber et al., 2011).

Yan reported for the first time that BRD4 is upregulated in bladder cancer tissues, and its high expression is closely associated with a more malignant clinical feature and poor patient prognosis (Yan et al., 2014). Recently, Wu revealed BRD4 as a novel promising target for pharmacologic treatment against bladder cancer and reported that BRD4 regulates proliferation and apoptosis of bladder cancer cells by positively regulating EZH2 transcription through upregulation of c-MYC (Wu et al., 2016). The Liu group showed that BRD4 promoted the migration, invasion, and DDP (cisplatin) resistance of BCa cells (Liu et al., 2022b). Multiple BRD4 protein small-molecule inhibitors, such as JQ1, have been developed and have shown promising anticancer results in experimental and clinical cancer studies (White et al., 2019). However, limitations of these BRD4 inhibitors were also displayed (Zhang et al., 2022). Some studies have shown that the accumulation of BRD4 protein is a key factor which confers resistance to BET inhibitors (Janouskova et al., 2017; Jin et al., 2018).

Recently, a target protein degradation strategy based on the concept of proteolysis targeting chimeric (PROTAC) molecules has been developed and has received increasing attention in the past decade (Neklesa et al., 2017; Burslem and Crews, 2020). These are heterobifunctional small molecules consisting of two ligands linked by a linker: one ligand binds to the protein of interest while the other binds to an E3 ubiquitin ligase, leading to ubiquitination and degradation of the target protein by the ubiquitin-proteasome system (UPS) (Békés et al., 2022). The novel pan BET degrader, QCA570 exerts potent degradation efficiency on BET proteins in leukemia cells and achieved complete and durable tumor regression in xenograft models at well-tolerated dose-schedules (Qin et al., 2018). In this study, we tested the potential anticancer activity and underlying signalling mechanisms of QCA570 on bladder cancer cells.

## Materials and methods

### Reagents

QCA570 was synthesized as reported previously (Qin et al., 2018). JQ1, MLN4924 and MG132 were purchased from Med Chem Express (Monmouth Junction, NJ).

### Cell lines

The human bladder cancer cell lines T24 and SV-HUC-1 was purchased from Procell Life Science & Technology Co., Ltd. (Wuhan, China). The 5637, J82, UMUC-3 and EJ-1 cells were obtained from Yuhuangding Hospital (Yantai, China). 5637, EJ-1, UM-UC-3 cells were cultured in RPMI1640 media (Procell, PM150110); J82 cells were cultured in MEM (NEAA) media (Procell, PM150410), and T24 cells were cultured in McCoy’s 5A media (PM150710). A total of 10% FBS (Gibco,1099141C) and 1% antibiotics (penicillin and streptomycin, Procell, PB180120) were added to the basic media. All the cells were cultured at 37°C with 5% CO_2_.

### Western blotting

Cells were lysed in RIPA lysis buffer (Beyotime Biotechnology, Jiangsu, China) with protease inhibitor (Roche, 11697498001) and phosphatase inhibitor (Roche, 4906837001) for 10 min on ice and shaken every 5 min on a vortex mixer for 30 s. Lysates were centrifuged at 12,000 rpm at 4°C for 10 min and the supernatant fraction was retained. Protein concentrations were quantified with BCA Protein Assay Kit (Thermo Fisher Scientific, A53226). Protein samples were separated *via* 10% SDS-PAGE and then transferred onto PVDF membrane. The membrane was blocked in 5% milk containing TBST (Tris-buffered Saline with Tween 20) at room temperature for 1 h, and this was followed by incubation with indicated primary antibodies overnight at 4°C. The membrane was then washed 3 times with TBST and incubated with secondary antibody for 1 h at room temperature before its development with the ECL kit (Thermo Fisher Scientific, 34,578). Primary antibodies to the following proteins were used: BRD4 (Bethyl Laboratories, A301-985A100), BRD3 (Bethyl Laboratories, A302-368A), BRD2 (Bethyl Laboratories, A302-583A), EZH2 (D2C9) (Cell Signaling Technology Inc, #5246), c-MYC (Abcam, ab32072), caspase-3 (Cell Signaling Technology Inc, #9662) GAPDH (ABclonal, AC033), β-Actin (ABclonal, AC006).

### Quantitative reverse transcriptase-polymerase chain reaction (qPCR)

Total RNA was extracted using the RNA-easy Isolation Reagent (Vazyme, R701) according to the manufacturer’s instructions. Complementary DNAs (cDNAs) were synthesized using HiScript III RT Super Mix for qPCR (+gDNA wiper) (Vazyme, R323). qPCR was performed using ChamQ Universal SYBR qPCR Master Mix (Vazyme, Q711) and detected with a LightCycler^®^ 96 Detection System (Roche, Switzerland). The PCR amplification was performed following the program: 95°C for 5 min of initial denaturation, then 40 cycles at 95°C for 30 s, 60°C for 30 s, and 72°C for 10 s, followed by a final melting curve step. The relative abundance of transcripts was calculated based on normalization to the GAPDH gene. The primers are shown in Table 1.

**TABLE 1:** The data sets collected from public GDSC database.

Primer	Sequence (5'—3')
GAPDH-F	TCAAGAAGGTGGTGAAGCAG
GAPDH-R	CGTCAAAG GTGGAGGAGTG
c-MYC-F	AGGGATCGCGCTGAGTATAA
c-MYC-R	TGCCTCTCGCTGGAATTACT
EZH2-F	CCCTGACCTCTGTCTTACTTGTGGA
EZH2-R	ACGT CAGATGGTGCCAGCAATA

### Cell viability by CCK-8 assay

5637, T24, EJ-1 at 3,000 cells/well and J82, UMUC-3 at 5,000 cells/well were seeded into 96-well plates. Cell viability and proliferation were measured with CCK-8 (Biosharp, BS350) at designated times after drug treatment. Briefly, 10 µL of CCK-8 solution was added to each well and incubated at 37°C for 2 h before reading the absorbance at 450 nm. The results were analyzed using GraphPad software. All experiments were repeated at least three times.

### Wound healing assay

Collect cells, seed them in 12-well plates, culture until the cells are completely confluent, and use a 200 μL sterilized pipette tip to make scratches perpendicular to the bottom of the culture dish. The original medium was discarded, washed twice with PBS, and the medium containing the compound was added again, and cultured in the incubator for 48 h. They were placed under a microscope and photographed at 8, 12, 24, and 48 h respectively.

### Flow cytometry assays for the cell cycle

Cells were harvested two days after treatment with compounds or DMSO, washed twice with PBS, and fixed with 70% ethanol overnight at 4°C. Then cells were centrifuged at 3,000 rpm for 5 min, washed twice with PBS, and resuspended in PBS. After incubating with 20 μg/mL RNase A (Solarbio Beijing, China), cells were stained with 20 μg/mL propidium iodide (PI) (Solarbio Beijing, China). A Beckman Coulter flow cytometer was used for detection and FlowJo-V10 software was used to analyze the data.

### Annexin V-FITC/PI apoptosis assay by flow cytometry

We harvested cells after a 2-day treatment with compounds or DMSO and washed them twice with cold PBS before resuspending them in 1x binding buffer. An Annexin V-FITC/PI Apoptosis Assay Kit (abs50001, Absin) was used to stain cells. After incubation for 15 min in the dark, we analyzed cell apoptosis on a flow cytometer (Beckman Coulter). Data were analyzed with FlowJo-V10 software.

### Terminal deoxyribonucleotide transferase-mediated dUTP nick-end labeling and staining

For this experiment, 5 × 10^5^ cells were grown on coverslips. Cell apoptosis was determined using the one-step TUNEL apoptosis assay kit (Roche, 12156792910) according to the manufacturer’s instructions. Following TUNEL staining, the slides were stained with DAPI (Solarbio Beijing, C0060) to highlight the nuclei with fluorescence. Slides were observed under an LSM 510 META Olympus Confocal Laser Scanning Microscope using 488-nm excitation and 530-nm emission, and the images were captured and quantified by image analysis software, ImageJ.

### Online analysis

The BRD4 and EZH2 gene expression across tumor and normal samples was analyzed online from TCGA clinical data on the UALCAN database (ualcan.path.uab.edu/home, accessed on may 3, 2022). TPM (transcripts per million) values were employed for the generation of boxplots and to estimate the significance between groups by *t*-test. Besides, BRD4 and EZH2 gene expression data were also downloaded from the publicly available GEO website (Home - GEO - NCBI (nih.gov), accessed on may 3, 2022) and included GSE48276 and GSE13507 datasets containing the raw RNA microarray data of 208 samples, comprised of 43 control and 165 BC patients. The Kaplan–Meier survival analysis was generated from the OncoLnc database (OncoLnc, accessed on May 3, 2022) which performs in-depth analyses of TCGA gene expression data. A Log-rank test was used for survival comparison.

## Results

### BRD4 is overexpressed in patients with BC and is associated with poor prognosis

**FIGURE 1:**
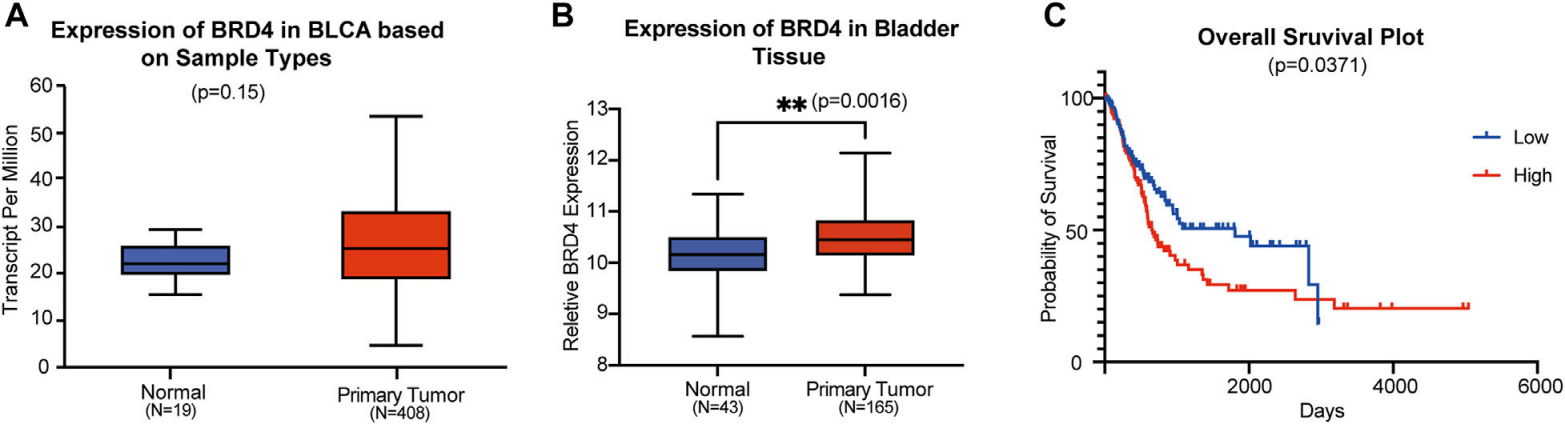
Association of BRD4 expression and bladder cancer risk **(A)** The BRD4 mRNA expression level of bladder tissues were analyzed in TGCA cohorts. The *p*-value was examined by Mann–Whitney U-test. **(B)** The relative expression level of BRD4 was determined in GEO database (GSE48276 and GSE13507). The remove Batch Effect function in the limma package was used to remove batches. The *p*-value was examined by Welch’s *t*-test. **(C)** The risk of BRD4 expression for survival analysis in 240 well-defined BC cases derived from TCGA databases was performed by the Kaplan-Meier plotter online. The *p*-value was calculated by a log-rank test.

To explore the role of BRD4 in BC progression, we analyzed the mRNA expression data from the TCGA database. In bladder tissues from 408 patients, BRD4 was upregulated compared with normal samples (Figure 1A). Data from the GEO website further confirmed that BRD4 was overexpressed in BC tissues (Figure 1B) from these patients. Kaplan-Meier survival analysis of 240 well-defined BC cases derived from the TCGA database showed that high BRD4 expression was associated with poor overall survival (OS) of BC patients (Figure 1C). These results showed the association between BRD4 expression with BC risk and suggest that BRD4 may be involved in a potential therapy target for BC patients.

### QCA570 is a potent BET degrader in cell lines of BC model

**FIGURE 2:**
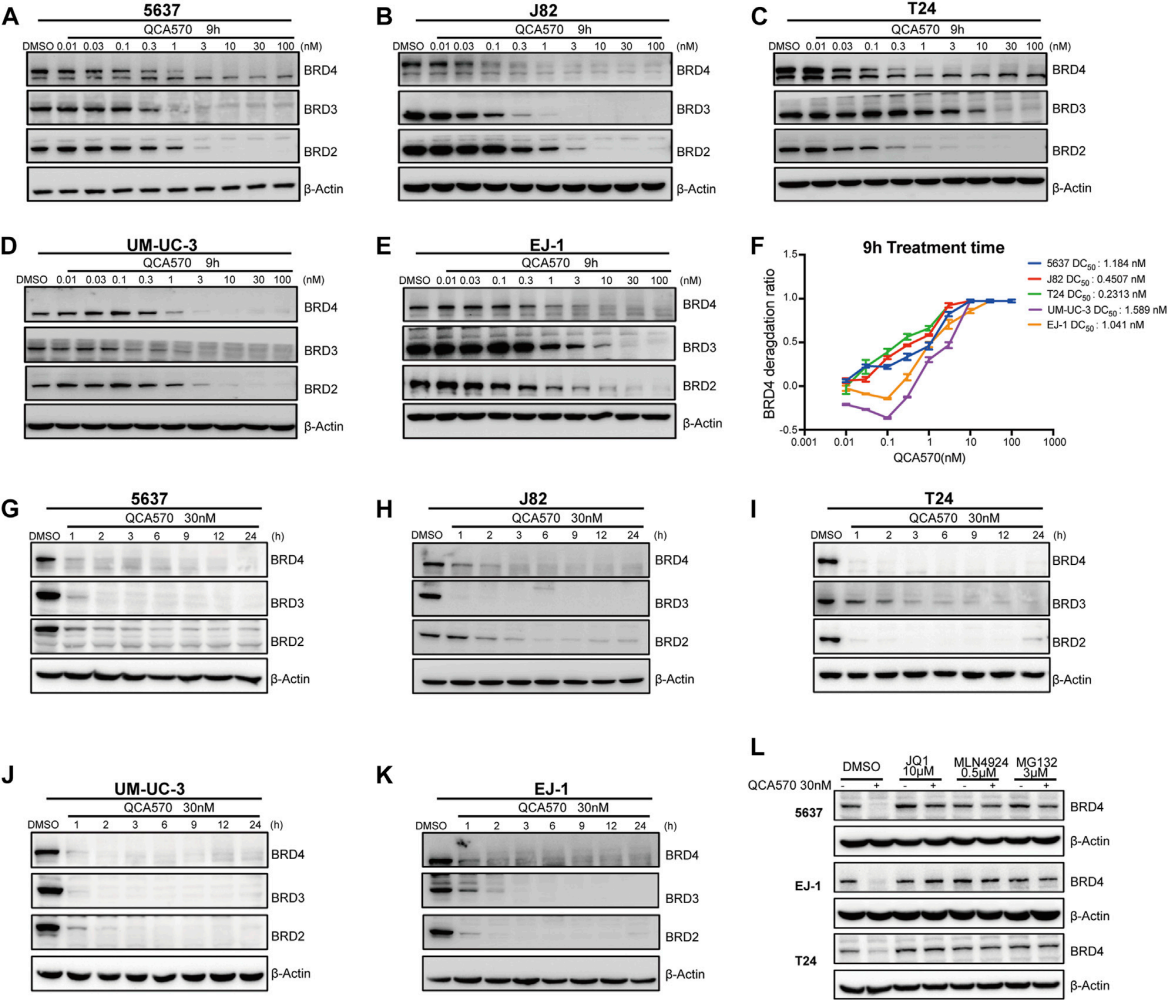
QCA570 potently induces BRD4 degradation in multiple BC cell lines **(A–F)** with a treatment of QCA570 at varied concentrations for 9 h in BC cells, the BET family proteins were detected by Western blotting. GAPDH was used as loading control. Protein levels were quantified with ImageJ and the degradation curve was plotted with GraphPad Prism 9. **(G–K)** BC cells were treated with 30 nM of QCA570 for indicated time points. BET family protein levels were determined by Western blotting and GAPDH was used as loading control. **(L)** Cells were pretreated with BETi JQ1, neddylation activating E1 enzyme inhibitor MLN4924 and proteasome inhibitor MG132 for 6 h, followed by treatment of QCA570 for a further 3 h. BRD4 protein levels were detected by Western blotting and β-Actin was used as a loading control.

The PROTAC molecule QCA570 (C_39_H_33_N_7_O_4_S, CAS number 2207569-08–0) developed by Qin et al. showed effective degradation of BET proteins BRD2, BRD3, and BRD4 at low picomolar concentrations and showed more potent proliferation inhibitory activity than other BET degraders such as dBET1, ARV-825, ARV-771, and ZBC260 in human leukemia cell lines. The degradation ability of QCA570 was also validated by Liu et al. in human lung cancer cells. In this study, we evaluated the activity of QCA570 on BC cell lines. As shown in Figures 2A-F, QCA570 induces degradation of BET proteins BRD2, BRD3 and BRD4 in a dose-dependent manner with a treatment of 9 h. BRD4 at 3 nM was largely depleted in each of these five BC cell lines with a DC_50_ around 1 nM. We next explored the degradation kinetics of QCA570 at 30 nM concentration at various duration times ranging from 1 to 24 h (Figures 2G-K). The BRD4 degradation peaked within 1 h in 5637, T24, UM-UC-3 cells and within 3 h in J82 and EJ-1 cell lines. A time-dependent degradation was also observed with BRD2 and BRD3 proteins.

Based on its PROTAC design, QCA570 forms a ternary complex dependent upon its binding to the target protein and E3 ubiquitin ligase CRBN. Similarly, QCA570 should also be proteasome-dependent. The mechanism used by QCA570 for the degradation of BET proteins was previously validated in RS4; 11 cells. In our study, we also confirmed the degradation mechanism in BC cells (Figure 2L). The Western blotting (WB) result showed that BRD4 degradation was almost completely abolished by pre-treatment with the proteasome inhibitor MG-132, the E1 neddylation inhibitor MLN4924, and the BRD4 ligand, JQ1 in BC cells. These data together provide evidence pertaining to the mechanism for ligands and proteasome-dependent degradation of BET proteins, consistent with its PROTAC character.

### QCA570 suppresses the expression of BRD4 target genes in BC cells

**FIGURE 3:**
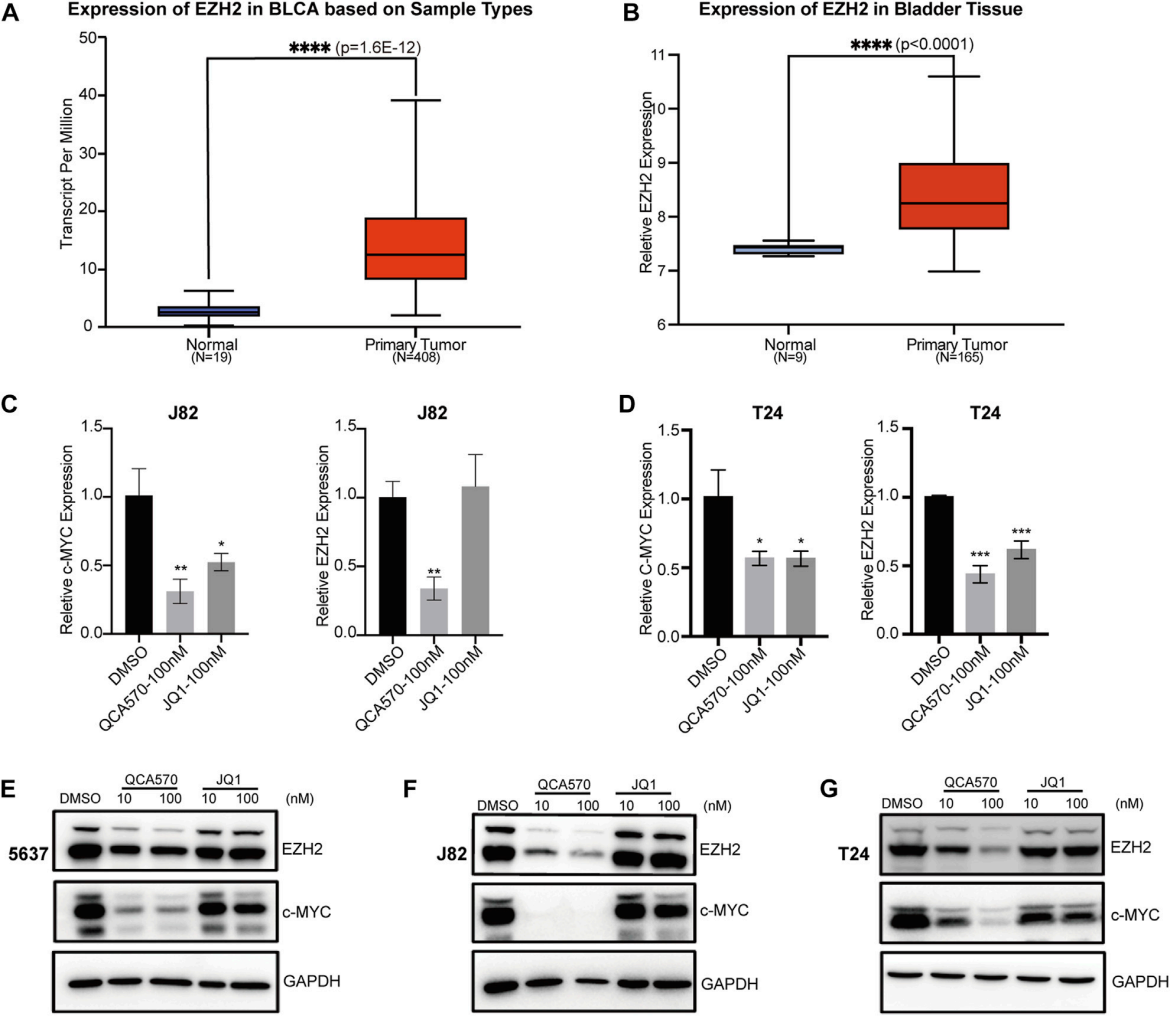
BRD4 target genes are downregulated by QCA570 **(A)** The EZH2 mRNA expression level of bladder tissues was analyzed in TGCA cohorts. The *p*-value was examined by the Mann–Whitney U-test. **(B)** The relative expression level of EZH2 was determined in GEO database (GSE13507). Using the remove Batch Effect function in the limma package to removes batches. The *p*-value was examined by Welch’s *t*-test. **(C, D)** RT-qPCR analysis of c-MYC and EZH2 gene expression in J82 and T24 cells. Values are the means ± SEM, *p < 0.05, **p < 0.01, ***p < 0.001, two-tailed Student’s t-test. **(E–G)** The protein level of c-MYC and EZH2 were detected by Western blotting. GAPDH was used as loading control.

Previous studies have reported that BET inhibition blocks expression of certain key oncogenes, such as c-MYC in many cancers (Filippakopoulos et al., 2010; Sahai et al., 2016). The Jiang group recently showed that BRD4 can promote EZH2 gene transcription through upregulation of c-MYC which functions as a transcription factor supporting BC progression (Xie et al., 2020; Wu et al., 2016). The mRNA expression data from TCGA and GEO database shows that EZH2 is upregulated in BC tissues, which suggests that the EZH2 gene is significantly associated with BC cancer risk (Figure 3A, B). To investigate whether BRD4 degradation down-regulates the expression level of EZH2, we determined the mRNA levels of these two genes in BC cell lines J82 and T24. In response to treatment with QCA570, the expression of c-MYC and EZH2 was significantly reduced with BRD4 degradation while the BET inhibitor JQ1 showed less suppression activity (Figures 3C, D). The protein level was also consistent with the qPCR results (Figures 3E-G).

### QCA570 inhibits the cancer phenotype of BC cells

**FIGURE 4:**
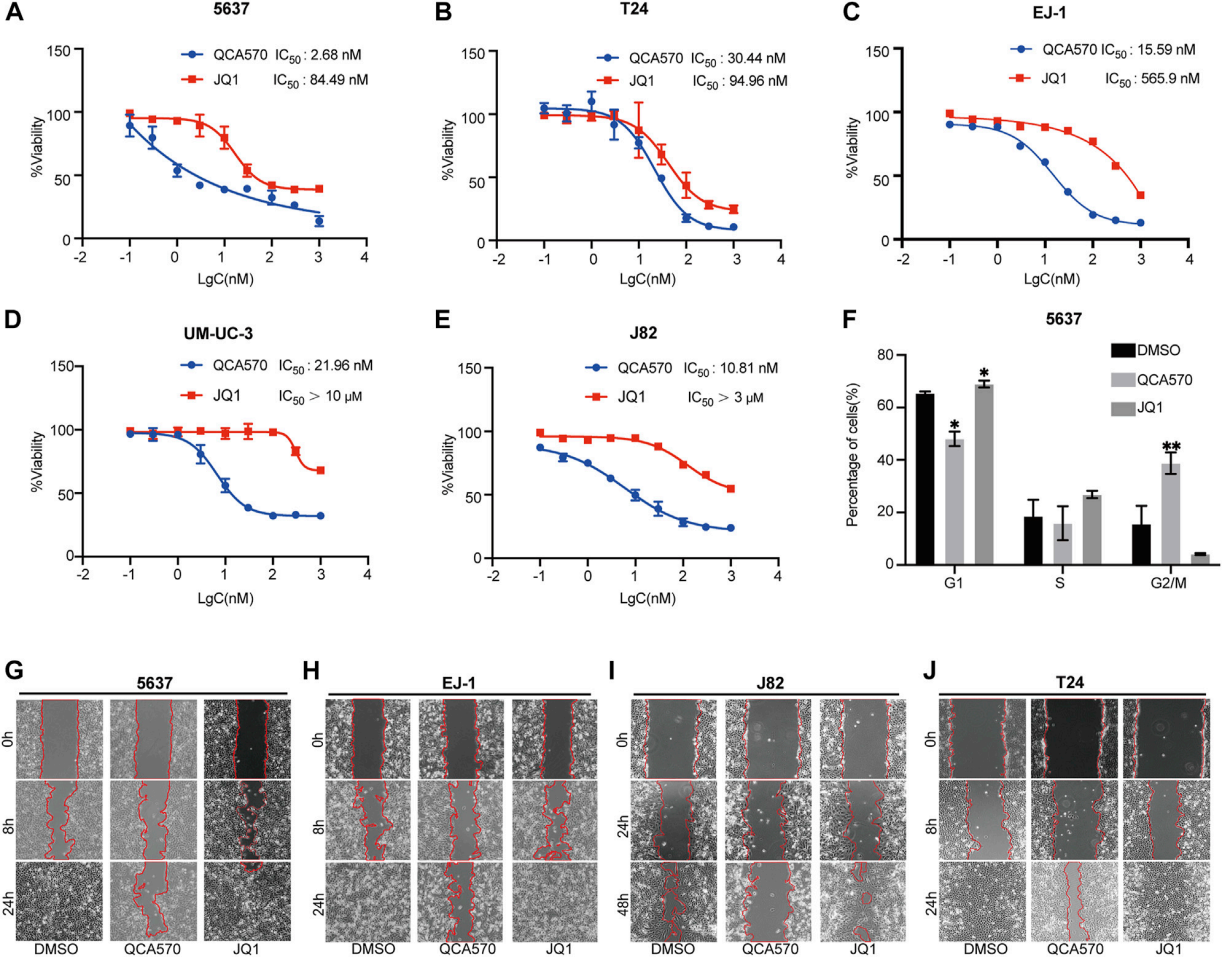
QCA570 treatment inhibits BC cell proliferation and migration **(A–E)** Antiproliferative effect of QCA570 or JQ1 in BC cell lines after 72 h treatment. Cell numbers were measured at the indicated time points with the CCK-8 assay and represented as absorbance at 450 nm. Values are the means ± SEM, *p < 0.05; **p < 0.01, two-tailed Student’s t-test. **(F)** 5637 cells were exposed to QCA570 (10 nM) or JQ1 (10 nM) for 72 h then collected for analysis using flow cytometry. Data was analyzed using Flowjo software and the quantified result was plotted with GraphPad Prism9.0. Values are the means ± SEM, *p < 0.05; **p < 0.01, two-tailed Student’s t-test. **(G-J)** Wound-healing assay was performed on 5637, EJ1, J82 and T24 cells with a treatment of QCA570 (100 nM) or JQ1 (100 nM) at indicated time points. The dotted line represents the wound healing boundary.

We next examined the antitumor effect of QCA570 on BC cell proliferation. As shown in Figure 4A-E, QCA570 effectively decreased the survival of the five BC cell lines tested, with IC_50_ values ranging from 2 to 30 nM. Notably, 5637 cells and J82 cells were particularly sensitive to QCA570 treatment, with IC_50_ values of 2.6 and 10.8 nM. In contrast, JQ1 is much less potent than QCA570 in each BC cell line tested. We also observed that QCA570 induced G2/M phase arrest of the cell cycle in 5637 cells (Figure 4F), further confirming the functional effect of QCA570. We investigated cell migration ability that represents another cancer-related feature which has been associated by wound healing assays with tumor metastasis. The results showed that with a 24 h treatment in 5637, EJ-1, J82 and T24 cells, QCA570 significantly suppressed cell migration ability (Figures 4G-J).

### QCA570 induces BC cell apoptosis

**FIGURE 5:**
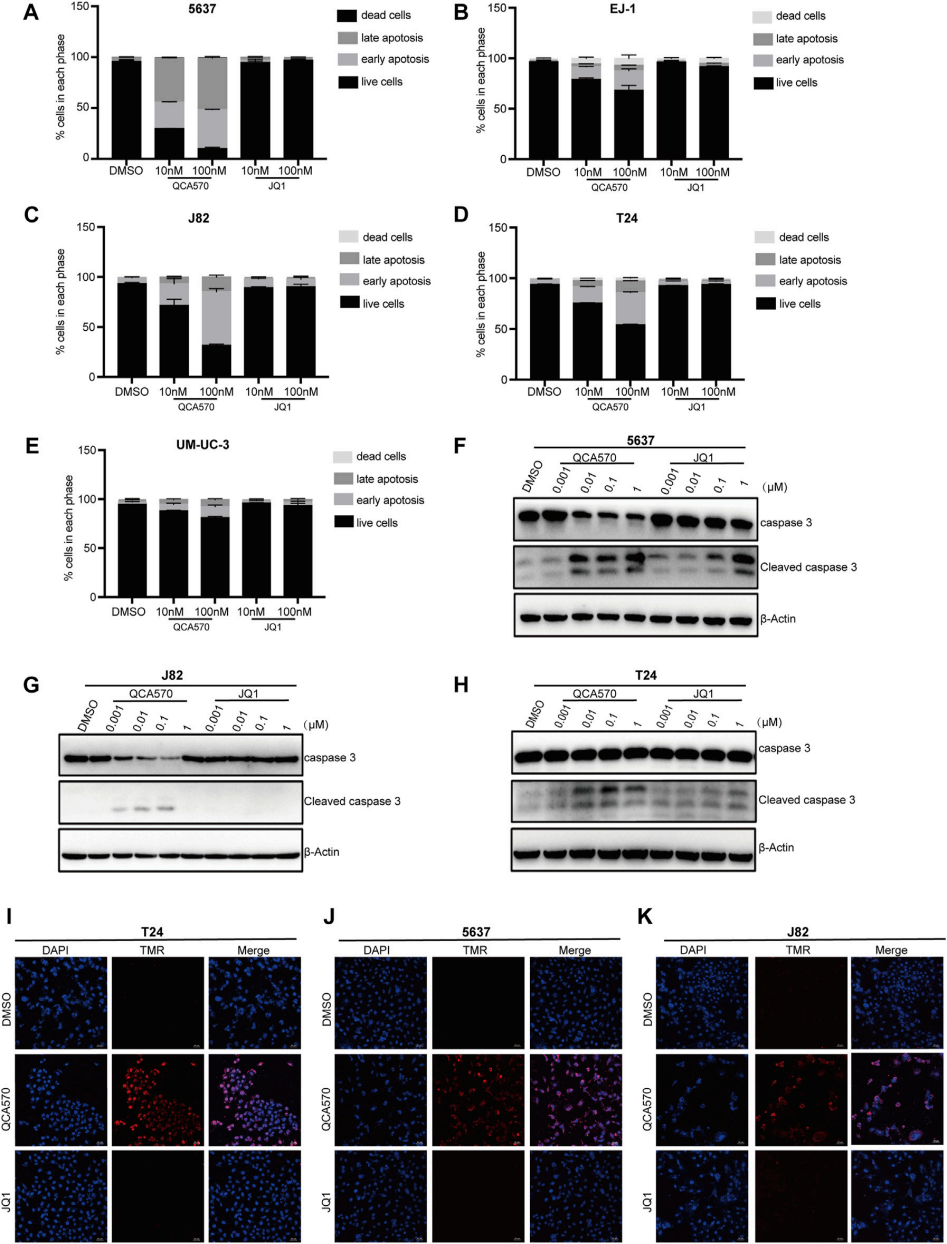
QCA570 induced cell apoptosis and cell cycle arrest **(A–E)** EJ-1, 5637, J82, UMUC-3 and T24 cells were treated with QCA570 or JQ1 for 72 h, and cell apoptosis was determined by Annexin V-FITC/PI staining and flow cytometry. Percentages of apoptosis cells were plotted using GraphPad Prism 9.0. Data were presented as means ± SEM. **(F–H)** 5637, J82 and T24 cells were treated with different concentrations of QCA570 or JQ1 for 72 h. Western blot analysis of the expression of cleaved caspase-3. **(I–K)** Apoptosis was analyzed by TUNEL staining. The red dots represented apoptotic cells, and DAPI (blue) indicated cell nuclei. Magnification, × 10.

To better understand the antiproliferation mechanism of QCA570, we investigated the effect of QCA570 on cell apoptosis in BC cell lines. Flow cytometry results demonstrated that QCA570 increased cell apoptosis in a dose-dependent manner (Figures 5A-E). In J82 and 5637 cell lines, apoptosis was induced in more than 50% of cells by QCA570 treatment. As a comparison, JQ1 had minimal effect on cell apoptosis. Consistent with the flow cytometry data, WB with cleaved Caspase-3 detection (Figures 5F-H) and a TUNEL assay of DNA break staining (Figures 5I-K) further confirmed that QCA570 induced greater cell apoptosis than in JQ1 in BC cells.

## Discussion

BRD4 is the most extensively studied member of the BET family, and has emerged as an attractive therapeutic target for cancer therapy. It is widely considered to be a driver of transcriptional activation and is involved in a variety of cancers by mediating the expression of oncogenes such as c-MYC, bcl-xL and bcl-6 (Lovén et al., 2013; Donati, Lorenzini, and Ciarrocchi 2018). It has been reported that BRD4 knockdown leads to cell cycle arrest, cell apoptosis and tumor growth inhibition in bladder cancer both *in vitro* and *in vivo*. In the current study, we observed after analyzing data from TGCA and GEO, that BRD4 was upregulated in BC tissues as compared to the corresponding normal bladder tissues. Furthermore, high expression of BRD4 was correlated with the poor prognosis of BC patients as shown by the Kaplan-Meier survival curve. These findings indicate that BRD4 might play a tumor-activator role in bladder cancer progression and targeting this protein could be an effective therapy for bladder cancer.

Recently, several potent small-molecule BRD4 inhibitors such as JQ1 and I-BET, which disrupt BET proteins binding to acetylated histones, have been discovered and have shown promising therapeutic potential in preclinical models of multiple cancers (Filippakopoulos et al., 2010; Filippakopoulos and Knapp 2014; Boi et al., 2015). However, due to drug resistance and lack of sustained transcriptional inhibition of targets, these agents showed limited clinical activity (Piya et al., 2019). For example, in Burkitt’s lymphoma cells, high concentrations and continuous exposure to BRD4 inhibitors were required to suppress the expression of c-MYC (Lu et al., 2015). In prostate cancer, mutated Speckle type POZ protein (SPOP) leads to impaired degradation and upregulation of BRD4 protein which confers intrinsic resistance to BET inhibitors (Janouskova et al., 2017; Dai et al., 2017; Zhang et al., 2017). In addition, several studies have revealed that BRD4 inhibitors cause the feedback elevation of BRD4 and less suppression of c-MYC expression (Fu et al., 2015; Hajmirza et al., 2018; White, Fenger, and Carson 2019). More recently, BET degraders, known as proteolysis targeting chimeric molecules (PROTACs), have been developed and exert more rapid, potent and durable inhibition of targets (Raina et al., 2016). The novel pan BET degrader, QCA570 showed more potent cell growth inhibitory activity in leukemia cell lines than previously published BET degraders such as dBET1,43 ARV-825,37 ARV-771,47 and ZBC26044 (Qin et al., 2018). The PROTAC molecule also displayed suppression of the survival of several human lung cancer cell lines with induction of apoptosis (C. Liu et al., 2022a). Therefore, in order to provide more potential therapies for bladder cancer, we employed this BET degrader, QCA570 to evaluate its antitumor effect on bladder cancer cells.

In our study, we observed a significant degradation of BET proteins induced by QCA570 in multiple BC cell lines and confirmed the PROTAC mechanism of QCA570 in BC cells. Wu has recently reported that BRD4 can positively regulate EZH2 gene expression through upregulation of c-MYC and that the BRD4/c-MYC/EZH2 axis plays a vital role in the regulation of bladder cancer progression. Therefore, we analyzed the expression of target genes under conditions of BRD4 depletion. Compared with BET inhibitor JQ1, QCA570 treatment resulted in much more suppression at the mRNA and protein levels of c-MYC and EZH2. Next, we explored the antitumoral activity of QCA570 on BC cells, as compared with JQ1. In multiple BC cell lines, QCA570 showed higher inhibition of cell proliferation and migration than JQ1. Meanwhile, we found that the G2/M cell cycle in 5637 cells was significantly arrested by QCA570, and further exploration with multiple detection methods showed that QCA570 efficiently induces cell apoptosis.

In conclusion, we have demonstrated that the high expression of BRD4 is correlated with the progression of BC patients and we tested the activity of the potent BRD4 degrader QCA570 on multiple BC cell models. We validated the suppression of c-MYC and EZH2 genes by QCA570 and its antitumor effect on BC cell lines. In view of the promising effects of the QCA570 against BC cells, we are providing a new approach to an effective therapy of human BC patients.

## Data Availability

Publicly available datasets were analyzed in this study. This data can be found here: https://www.ncbi.nlm.nih.gov/geo/ - GSE48276 and GSE13507.
