# Supplementary figures and images for: Lethal activity of BRD4 PROTAC degrader QCA570 against bladder cancer cells
Source: Front Chem. 2023 Jan 17;11:1121724. doi: 10.3389/fchem.2023.1121724 (PMC9887192; doi:10.3389/fchem.2023.1121724)

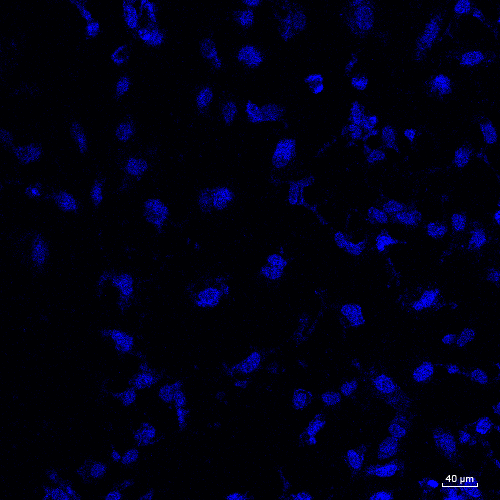

Supplement: Supplementary file 2 [file DataSheet11.ZIP › Terminal deoxyribonucleotide transferase-mediated dUTP nick-end labeling staining/5637/570-dapi.tif]

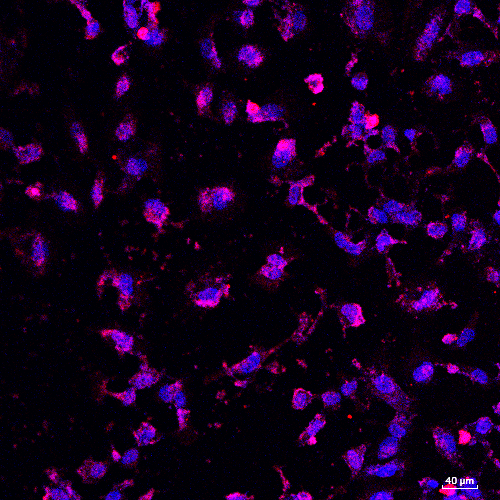

Supplement: Supplementary file 2 [file DataSheet11.ZIP › Terminal deoxyribonucleotide transferase-mediated dUTP nick-end labeling staining/5637/570-merge.tif]

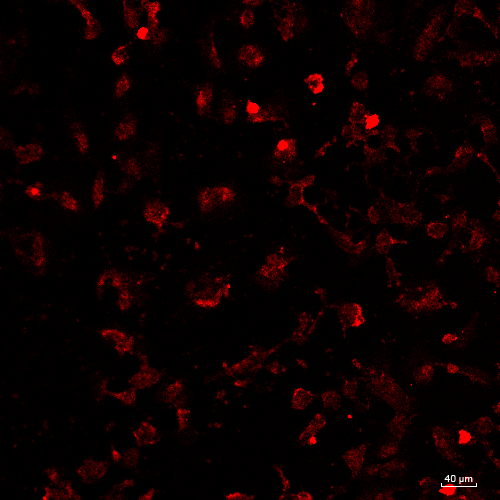

Supplement: Supplementary file 2 [file DataSheet11.ZIP › Terminal deoxyribonucleotide transferase-mediated dUTP nick-end labeling staining/5637/570-tunel.tif]

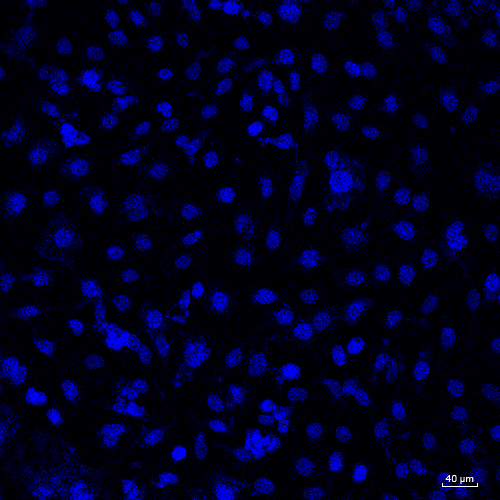

Supplement: Supplementary file 2 [file DataSheet11.ZIP › Terminal deoxyribonucleotide transferase-mediated dUTP nick-end labeling staining/5637/dmso-dapi.tif]

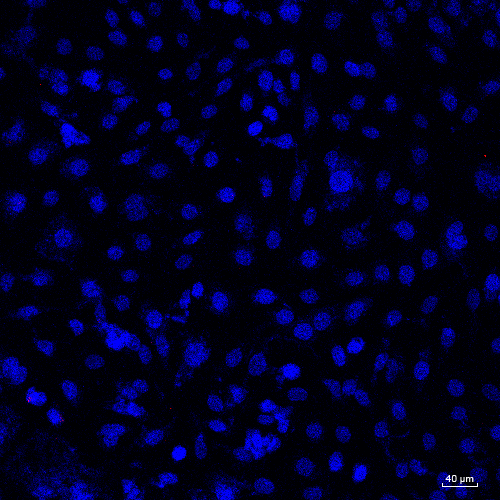

Supplement: Supplementary file 2 [file DataSheet11.ZIP › Terminal deoxyribonucleotide transferase-mediated dUTP nick-end labeling staining/5637/dmso-merge.tif]

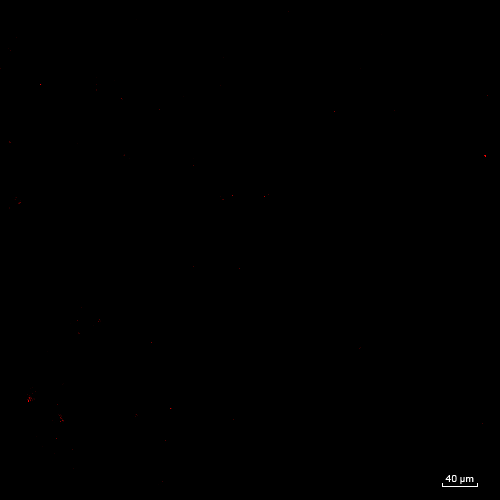

Supplement: Supplementary file 2 [file DataSheet11.ZIP › Terminal deoxyribonucleotide transferase-mediated dUTP nick-end labeling staining/5637/dmso-tunel.tif]

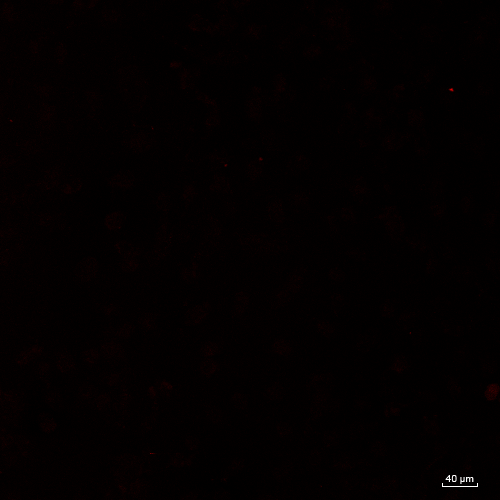

Supplement: Supplementary file 2 [file DataSheet11.ZIP › Terminal deoxyribonucleotide transferase-mediated dUTP nick-end labeling staining/5637/jq1-dapi.tif]

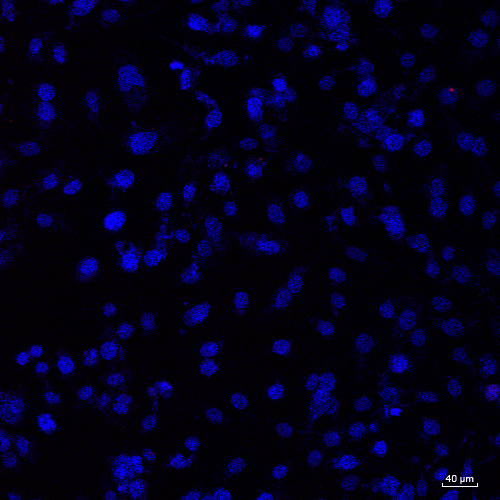

Supplement: Supplementary file 2 [file DataSheet11.ZIP › Terminal deoxyribonucleotide transferase-mediated dUTP nick-end labeling staining/5637/jq1-merge.tif]

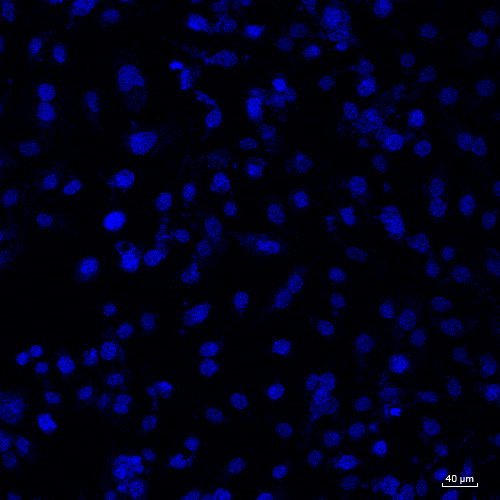

Supplement: Supplementary file 2 [file DataSheet11.ZIP › Terminal deoxyribonucleotide transferase-mediated dUTP nick-end labeling staining/5637/jq1-tunel.tif]

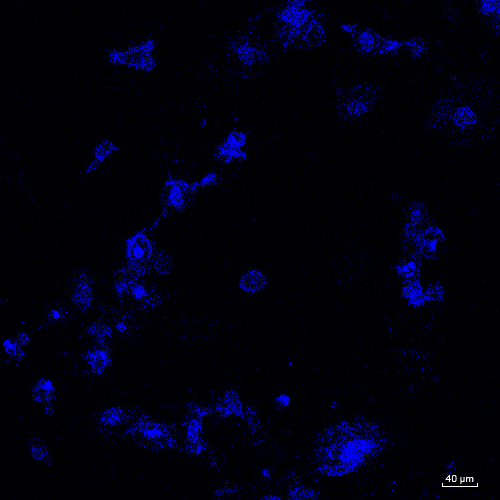

Supplement: Supplementary file 2 [file DataSheet11.ZIP › Terminal deoxyribonucleotide transferase-mediated dUTP nick-end labeling staining/J82/570-dapi.tif]

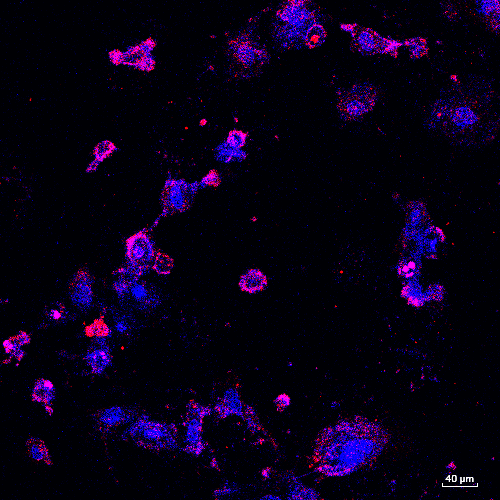

Supplement: Supplementary file 2 [file DataSheet11.ZIP › Terminal deoxyribonucleotide transferase-mediated dUTP nick-end labeling staining/J82/570-merge.tif]

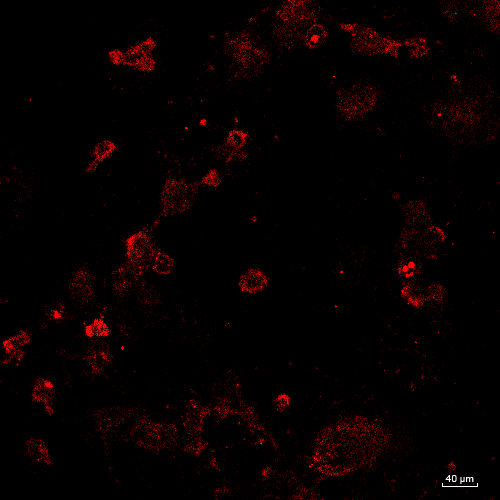

Supplement: Supplementary file 2 [file DataSheet11.ZIP › Terminal deoxyribonucleotide transferase-mediated dUTP nick-end labeling staining/J82/570-tunel.tif]

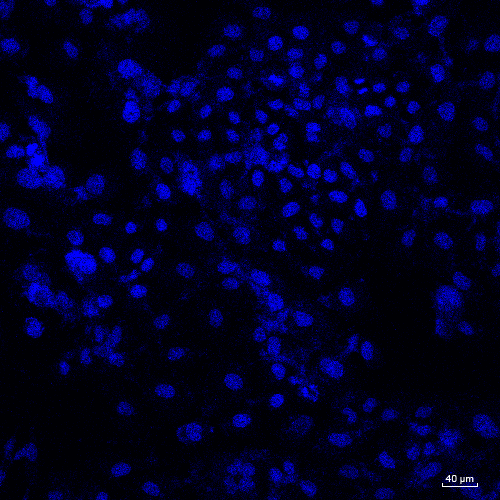

Supplement: Supplementary file 2 [file DataSheet11.ZIP › Terminal deoxyribonucleotide transferase-mediated dUTP nick-end labeling staining/J82/dmso-dapi.tif]

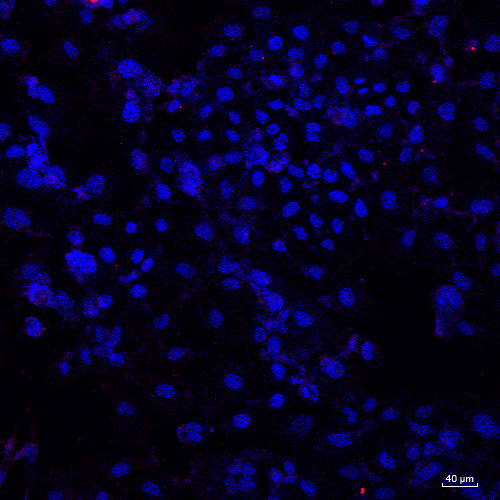

Supplement: Supplementary file 2 [file DataSheet11.ZIP › Terminal deoxyribonucleotide transferase-mediated dUTP nick-end labeling staining/J82/dmso-merge.tif]

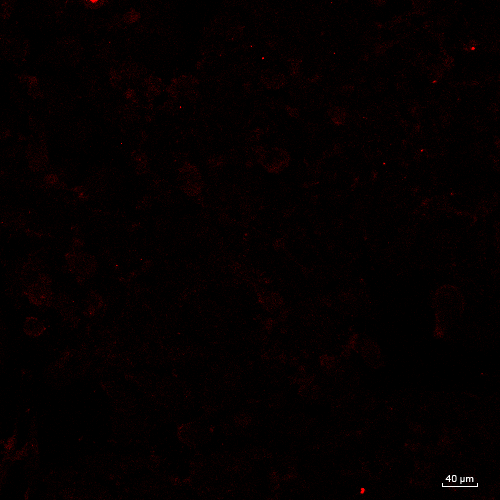

Supplement: Supplementary file 2 [file DataSheet11.ZIP › Terminal deoxyribonucleotide transferase-mediated dUTP nick-end labeling staining/J82/dmso-tunel.tif]

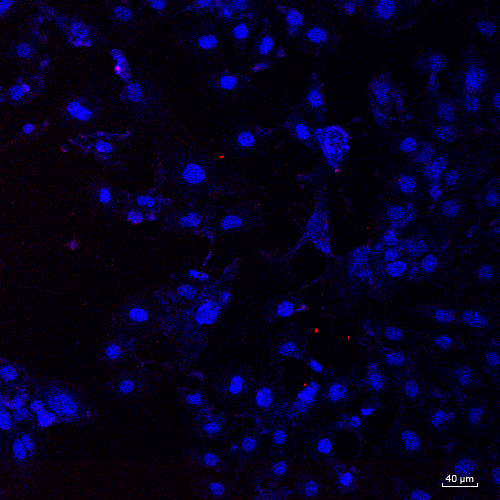

Supplement: Supplementary file 2 [file DataSheet11.ZIP › Terminal deoxyribonucleotide transferase-mediated dUTP nick-end labeling staining/J82/jq-merge.tif]

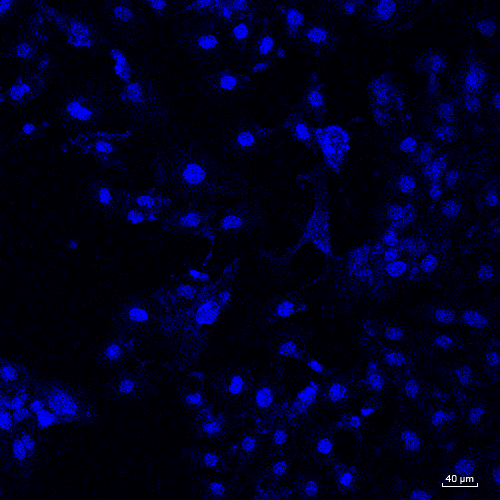

Supplement: Supplementary file 2 [file DataSheet11.ZIP › Terminal deoxyribonucleotide transferase-mediated dUTP nick-end labeling staining/J82/jq1-dapi.tif]

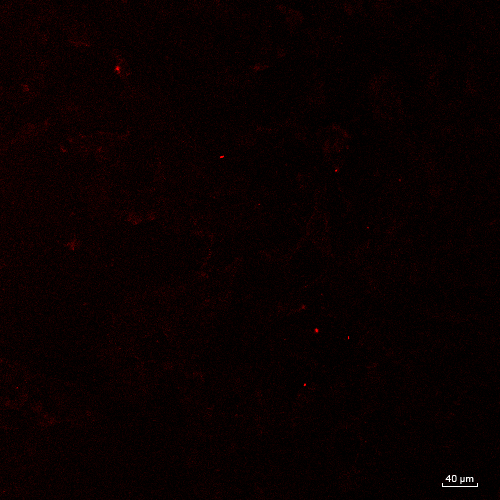

Supplement: Supplementary file 2 [file DataSheet11.ZIP › Terminal deoxyribonucleotide transferase-mediated dUTP nick-end labeling staining/J82/jq1-tunel.tif]

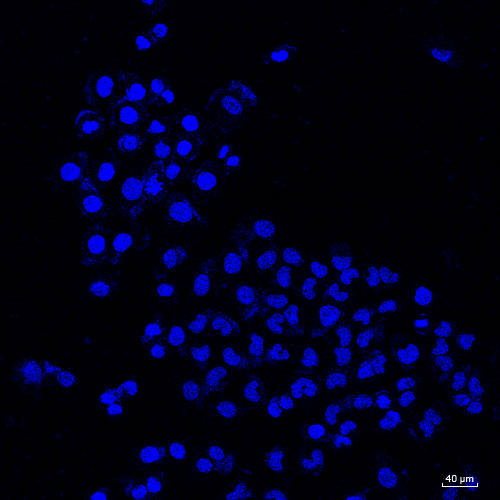

Supplement: Supplementary file 2 [file DataSheet11.ZIP › Terminal deoxyribonucleotide transferase-mediated dUTP nick-end labeling staining/t24/570-dapi.tif]

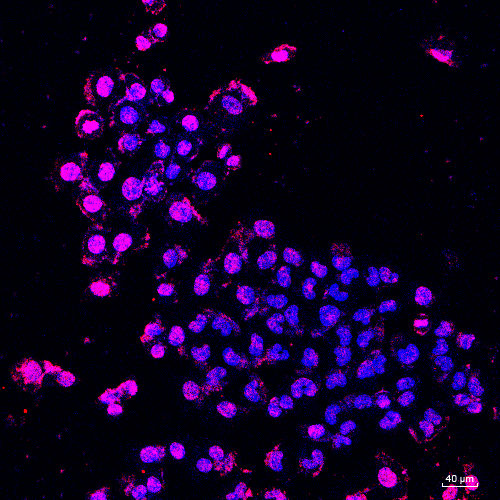

Supplement: Supplementary file 2 [file DataSheet11.ZIP › Terminal deoxyribonucleotide transferase-mediated dUTP nick-end labeling staining/t24/570-merge.tif]

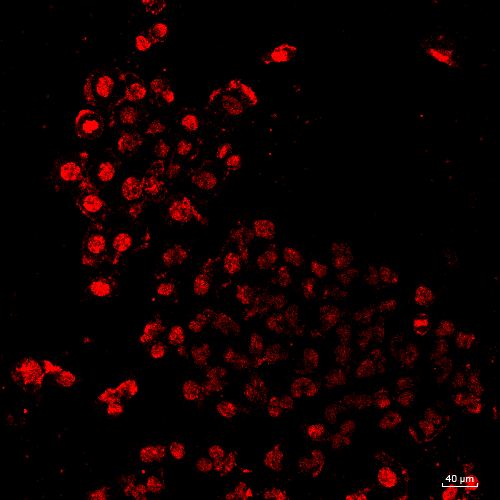

Supplement: Supplementary file 2 [file DataSheet11.ZIP › Terminal deoxyribonucleotide transferase-mediated dUTP nick-end labeling staining/t24/570-tunel.tif]

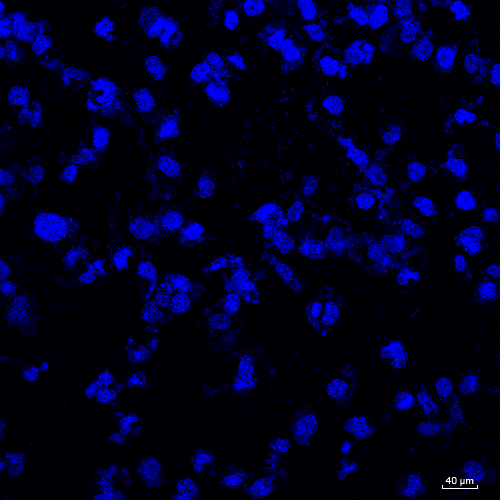

Supplement: Supplementary file 2 [file DataSheet11.ZIP › Terminal deoxyribonucleotide transferase-mediated dUTP nick-end labeling staining/t24/dmso-dapi.tif]

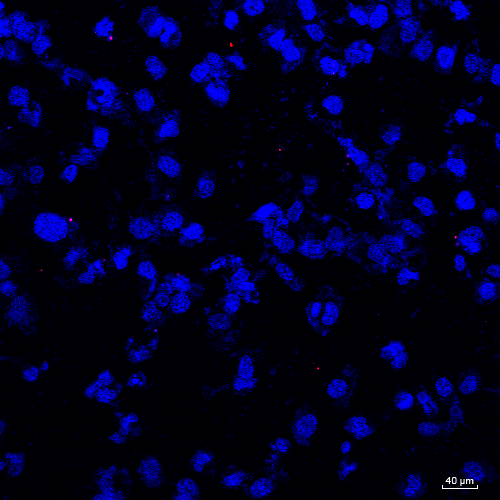

Supplement: Supplementary file 2 [file DataSheet11.ZIP › Terminal deoxyribonucleotide transferase-mediated dUTP nick-end labeling staining/t24/dmso-merge.tif]

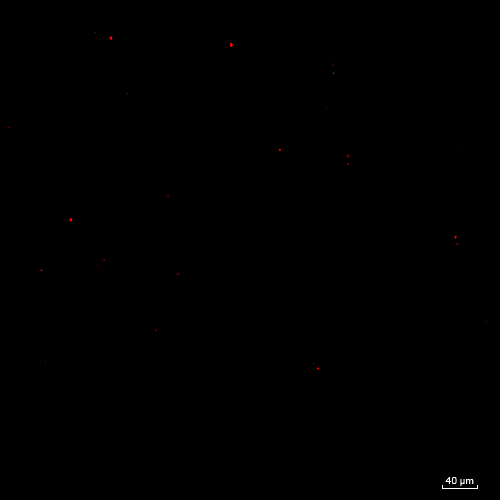

Supplement: Supplementary file 2 [file DataSheet11.ZIP › Terminal deoxyribonucleotide transferase-mediated dUTP nick-end labeling staining/t24/dmso-tunel.tif]

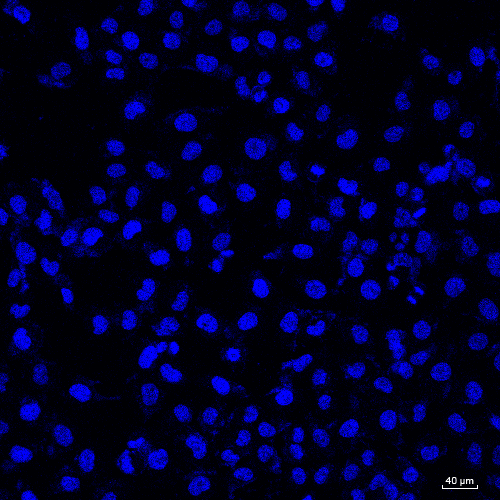

Supplement: Supplementary file 2 [file DataSheet11.ZIP › Terminal deoxyribonucleotide transferase-mediated dUTP nick-end labeling staining/t24/jq1-dapi.tif]

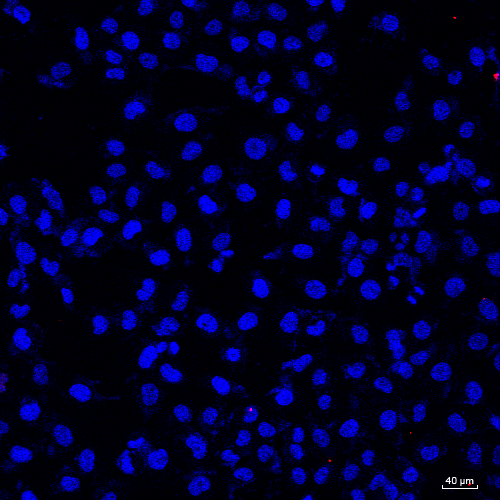

Supplement: Supplementary file 2 [file DataSheet11.ZIP › Terminal deoxyribonucleotide transferase-mediated dUTP nick-end labeling staining/t24/jq1-merge.tif]

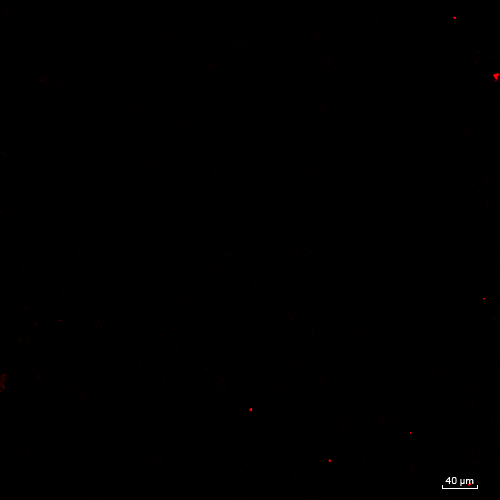

Supplement: Supplementary file 2 [file DataSheet11.ZIP › Terminal deoxyribonucleotide transferase-mediated dUTP nick-end labeling staining/t24/jq1-tunel.tif]

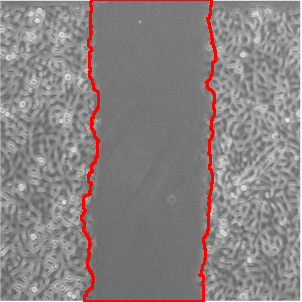

Supplement: Supplementary file 5 [file DataSheet4.ZIP › Fig4-G-J-wound healing assay/5637/Fig4-5637-570-10-0h.png]

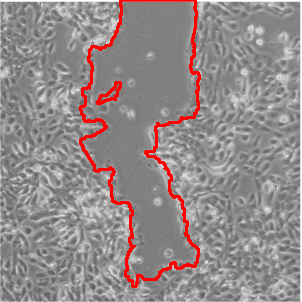

Supplement: Supplementary file 5 [file DataSheet4.ZIP › Fig4-G-J-wound healing assay/5637/Fig4-5637-570-10-24h.png]

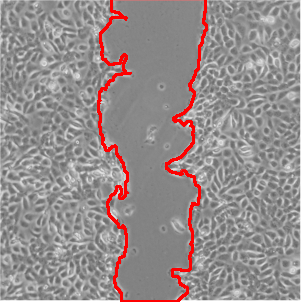

Supplement: Supplementary file 5 [file DataSheet4.ZIP › Fig4-G-J-wound healing assay/5637/Fig4-5637-570-10-8h.png]

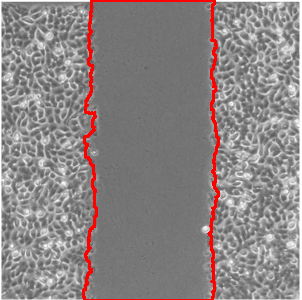

Supplement: Supplementary file 5 [file DataSheet4.ZIP › Fig4-G-J-wound healing assay/5637/Fig4-5637-DMSO-0.png]

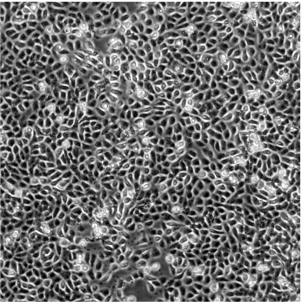

Supplement: Supplementary file 5 [file DataSheet4.ZIP › Fig4-G-J-wound healing assay/5637/Fig4-5637-DMSO-24h.png]

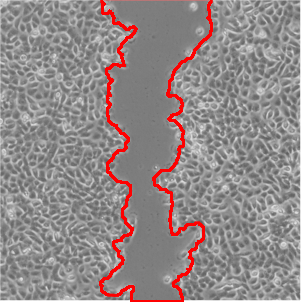

Supplement: Supplementary file 5 [file DataSheet4.ZIP › Fig4-G-J-wound healing assay/5637/Fig4-5637-DMSO-8h.png]

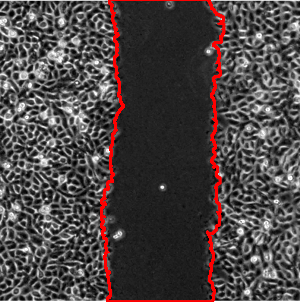

Supplement: Supplementary file 5 [file DataSheet4.ZIP › Fig4-G-J-wound healing assay/5637/Fig4-5637-JQ1-0.png]

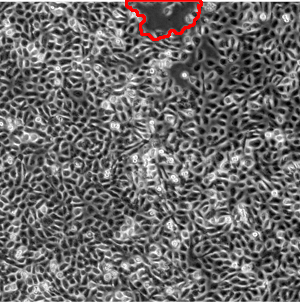

Supplement: Supplementary file 5 [file DataSheet4.ZIP › Fig4-G-J-wound healing assay/5637/Fig4-5637-JQ1-10-24h.png]

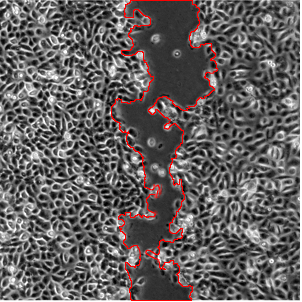

Supplement: Supplementary file 5 [file DataSheet4.ZIP › Fig4-G-J-wound healing assay/5637/Fig4-5637-JQ1-10-8h.png]

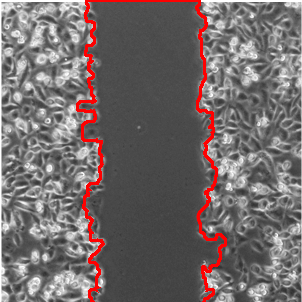

Supplement: Supplementary file 5 [file DataSheet4.ZIP › Fig4-G-J-wound healing assay/EJ-1/Fig4-EJ-1-570-100-0h.png]

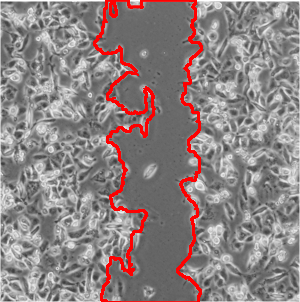

Supplement: Supplementary file 5 [file DataSheet4.ZIP › Fig4-G-J-wound healing assay/EJ-1/Fig4-EJ-1-570-100-24h.png]

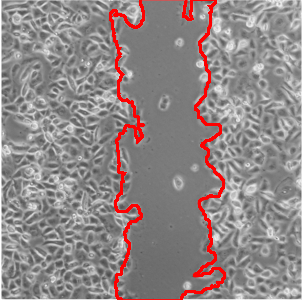

Supplement: Supplementary file 5 [file DataSheet4.ZIP › Fig4-G-J-wound healing assay/EJ-1/Fig4-EJ-1-570-100-8h.png]

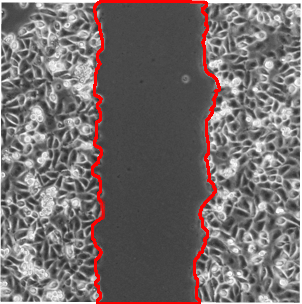

Supplement: Supplementary file 5 [file DataSheet4.ZIP › Fig4-G-J-wound healing assay/EJ-1/Fig4-EJ-1-DMSO-0.png]

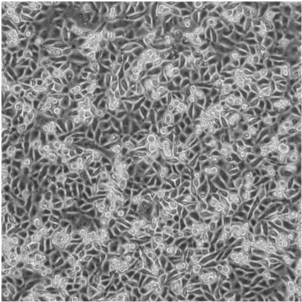

Supplement: Supplementary file 5 [file DataSheet4.ZIP › Fig4-G-J-wound healing assay/EJ-1/Fig4-EJ-1-DMSO-24h.png]

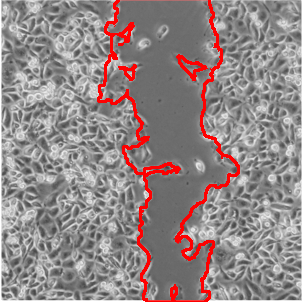

Supplement: Supplementary file 5 [file DataSheet4.ZIP › Fig4-G-J-wound healing assay/EJ-1/Fig4-EJ-1-DMSO-8h.png]

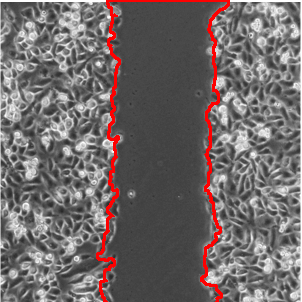

Supplement: Supplementary file 5 [file DataSheet4.ZIP › Fig4-G-J-wound healing assay/EJ-1/Fig4-EJ-1-JQ1-100-0h.png]

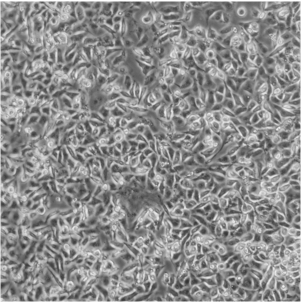

Supplement: Supplementary file 5 [file DataSheet4.ZIP › Fig4-G-J-wound healing assay/EJ-1/Fig4-EJ-1-JQ1-100-24h.png]

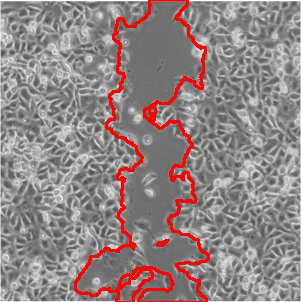

Supplement: Supplementary file 5 [file DataSheet4.ZIP › Fig4-G-J-wound healing assay/EJ-1/Fig4-EJ-1-JQ1-100-8h.png]

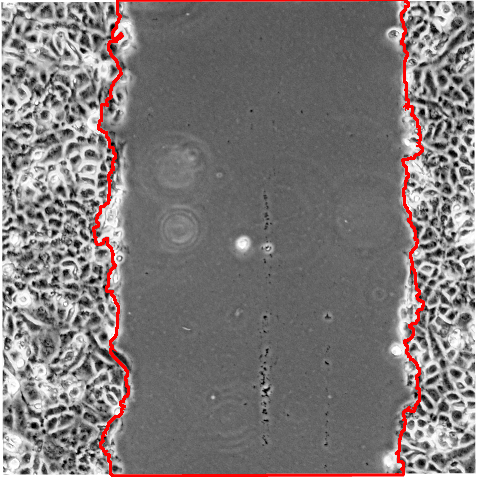

Supplement: Supplementary file 5 [file DataSheet4.ZIP › Fig4-G-J-wound healing assay/J82/Fig4-J82-570-100-0h.png]

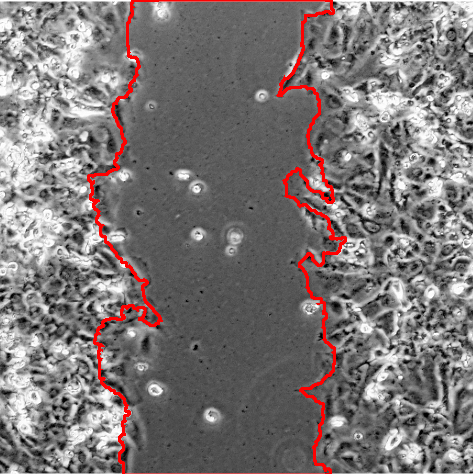

Supplement: Supplementary file 5 [file DataSheet4.ZIP › Fig4-G-J-wound healing assay/J82/Fig4-J82-570-100-24h.png]

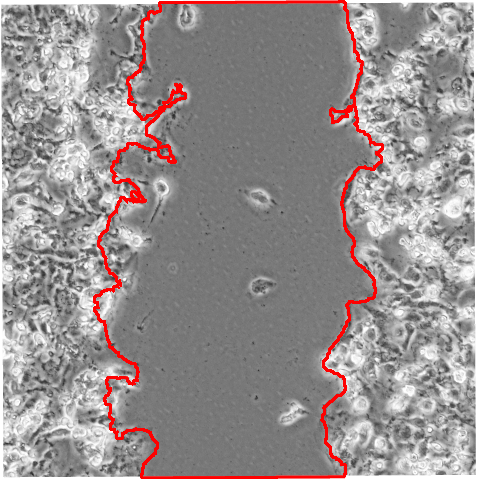

Supplement: Supplementary file 5 [file DataSheet4.ZIP › Fig4-G-J-wound healing assay/J82/Fig4-J82-570-100-48h.png]

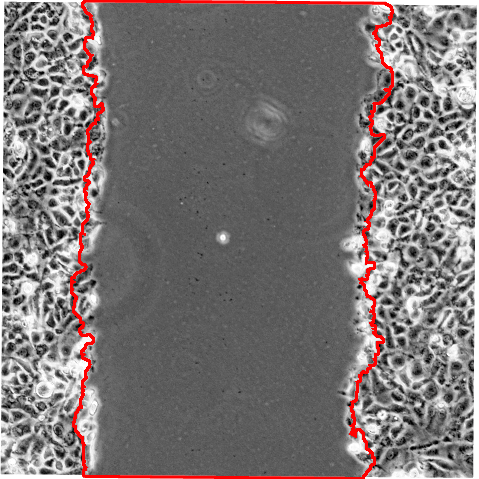

Supplement: Supplementary file 5 [file DataSheet4.ZIP › Fig4-G-J-wound healing assay/J82/Fig4-J82-DMSO-0h.png]

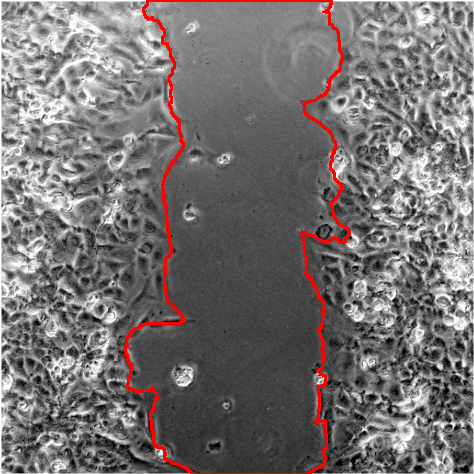

Supplement: Supplementary file 5 [file DataSheet4.ZIP › Fig4-G-J-wound healing assay/J82/Fig4-J82-DMSO-24h.png]

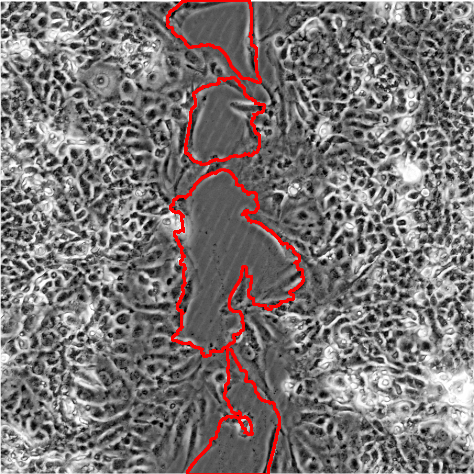

Supplement: Supplementary file 5 [file DataSheet4.ZIP › Fig4-G-J-wound healing assay/J82/Fig4-J82-DMSO-48h.png]

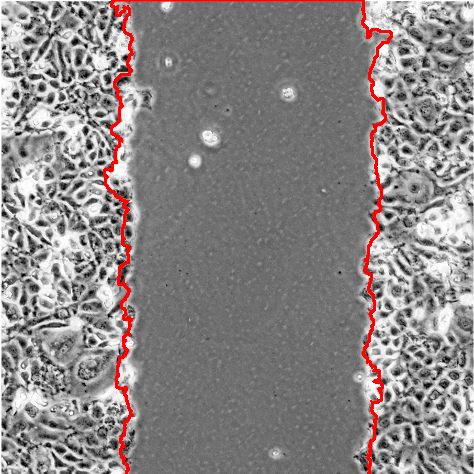

Supplement: Supplementary file 5 [file DataSheet4.ZIP › Fig4-G-J-wound healing assay/J82/Fig4-J82-JQ1-100-0h.png]

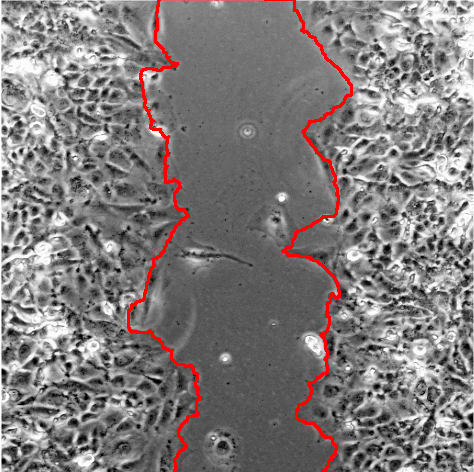

Supplement: Supplementary file 5 [file DataSheet4.ZIP › Fig4-G-J-wound healing assay/J82/Fig4-J82-JQ1-100-24h.png]

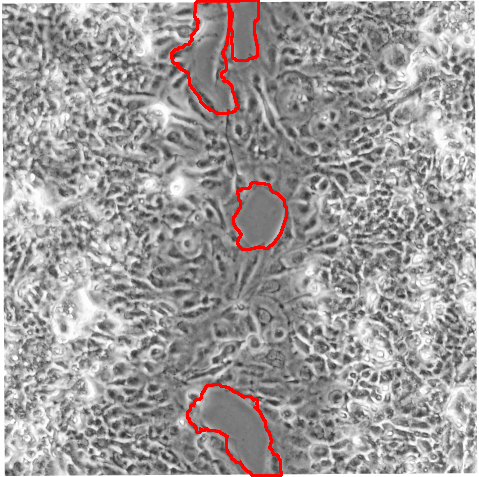

Supplement: Supplementary file 5 [file DataSheet4.ZIP › Fig4-G-J-wound healing assay/J82/Fig4-J82-JQ1-100-48h.png]

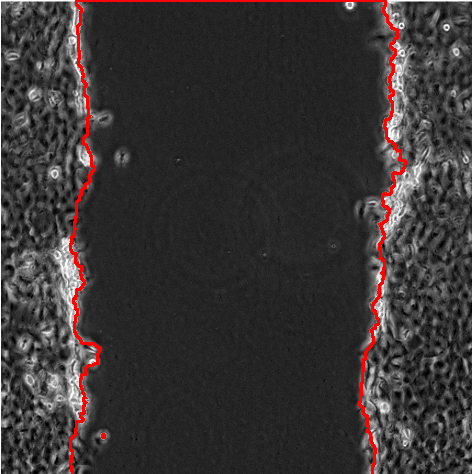

Supplement: Supplementary file 5 [file DataSheet4.ZIP › Fig4-G-J-wound healing assay/T24/Fig4-T24-570-100-0h.png]

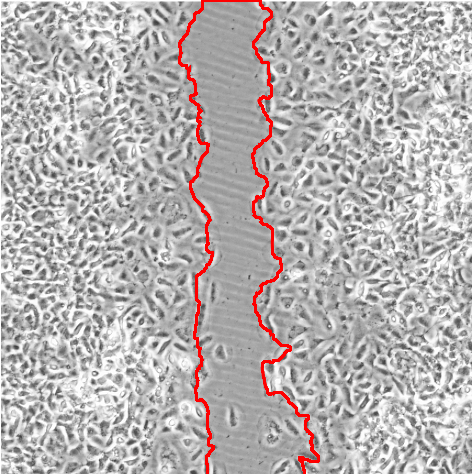

Supplement: Supplementary file 5 [file DataSheet4.ZIP › Fig4-G-J-wound healing assay/T24/Fig4-T24-570-100-24h.png]

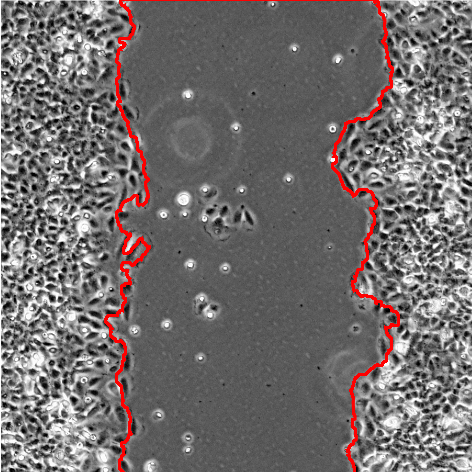

Supplement: Supplementary file 5 [file DataSheet4.ZIP › Fig4-G-J-wound healing assay/T24/Fig4-T24-570-100-8h.png]

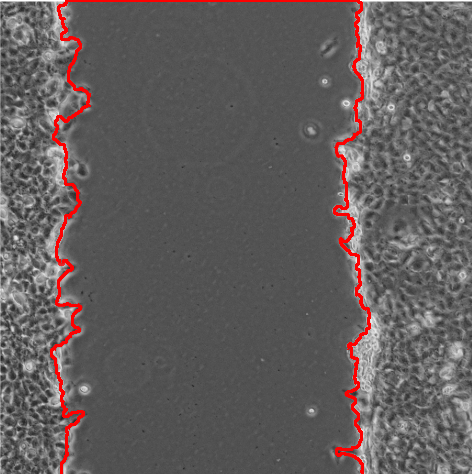

Supplement: Supplementary file 5 [file DataSheet4.ZIP › Fig4-G-J-wound healing assay/T24/Fig4-T24-DMSO-0h.png]

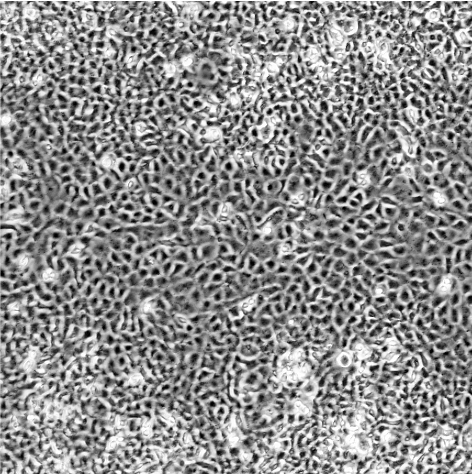

Supplement: Supplementary file 5 [file DataSheet4.ZIP › Fig4-G-J-wound healing assay/T24/Fig4-T24-DMSO-24h.png]

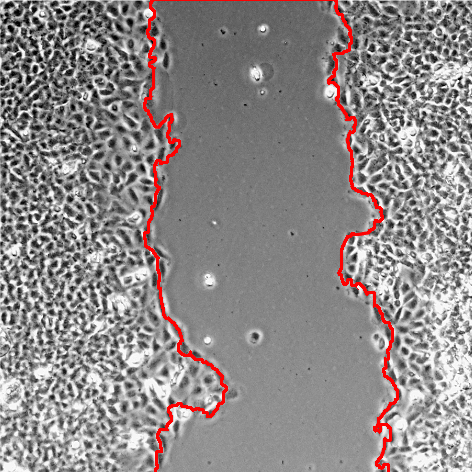

Supplement: Supplementary file 5 [file DataSheet4.ZIP › Fig4-G-J-wound healing assay/T24/Fig4-T24-DMSO-8h.png]

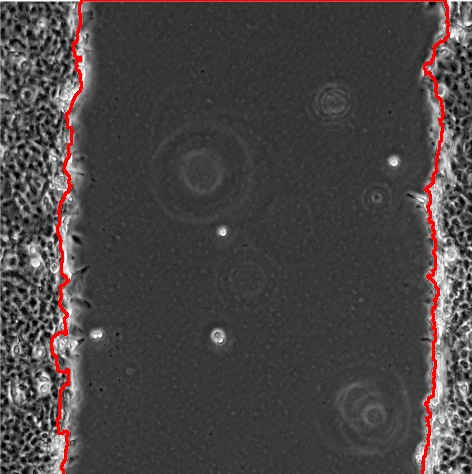

Supplement: Supplementary file 5 [file DataSheet4.ZIP › Fig4-G-J-wound healing assay/T24/Fig4-T24-JQ1-100-0h.png]

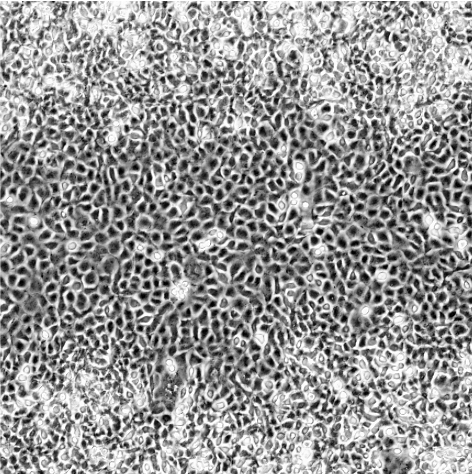

Supplement: Supplementary file 5 [file DataSheet4.ZIP › Fig4-G-J-wound healing assay/T24/Fig4-T24-JQ1-100-24h.png]

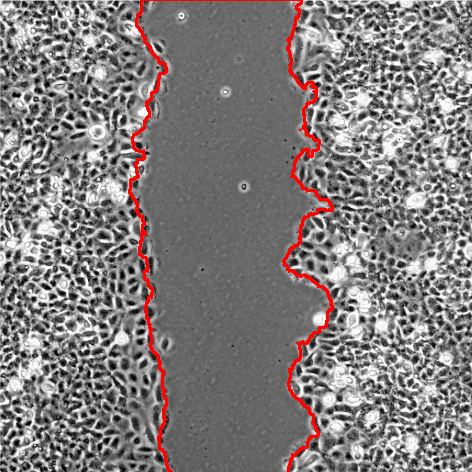

Supplement: Supplementary file 5 [file DataSheet4.ZIP › Fig4-G-J-wound healing assay/T24/Fig4-T24-JQ1-100-8h.png]

## Slide 1
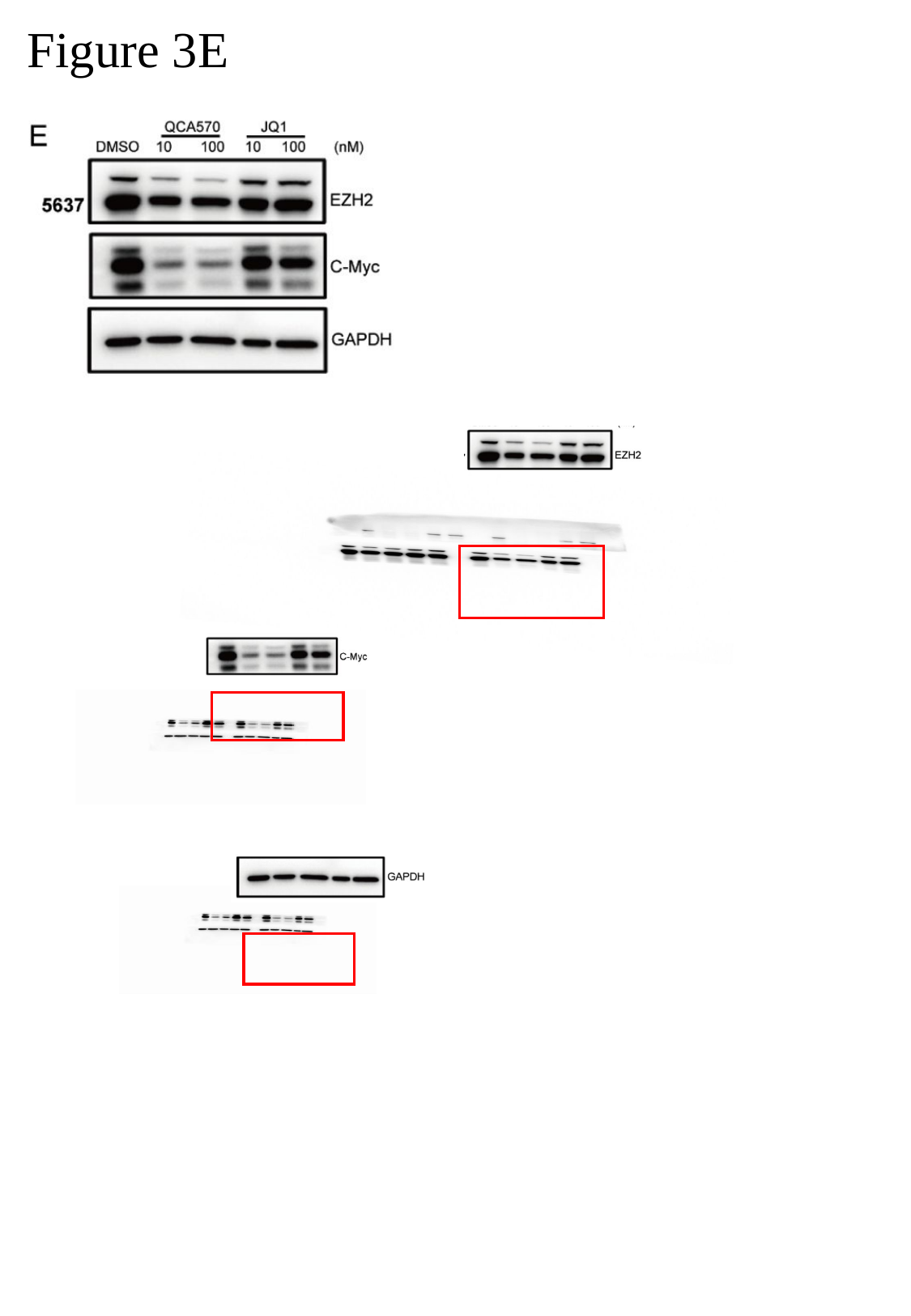

Figure 3E

## Slide 2
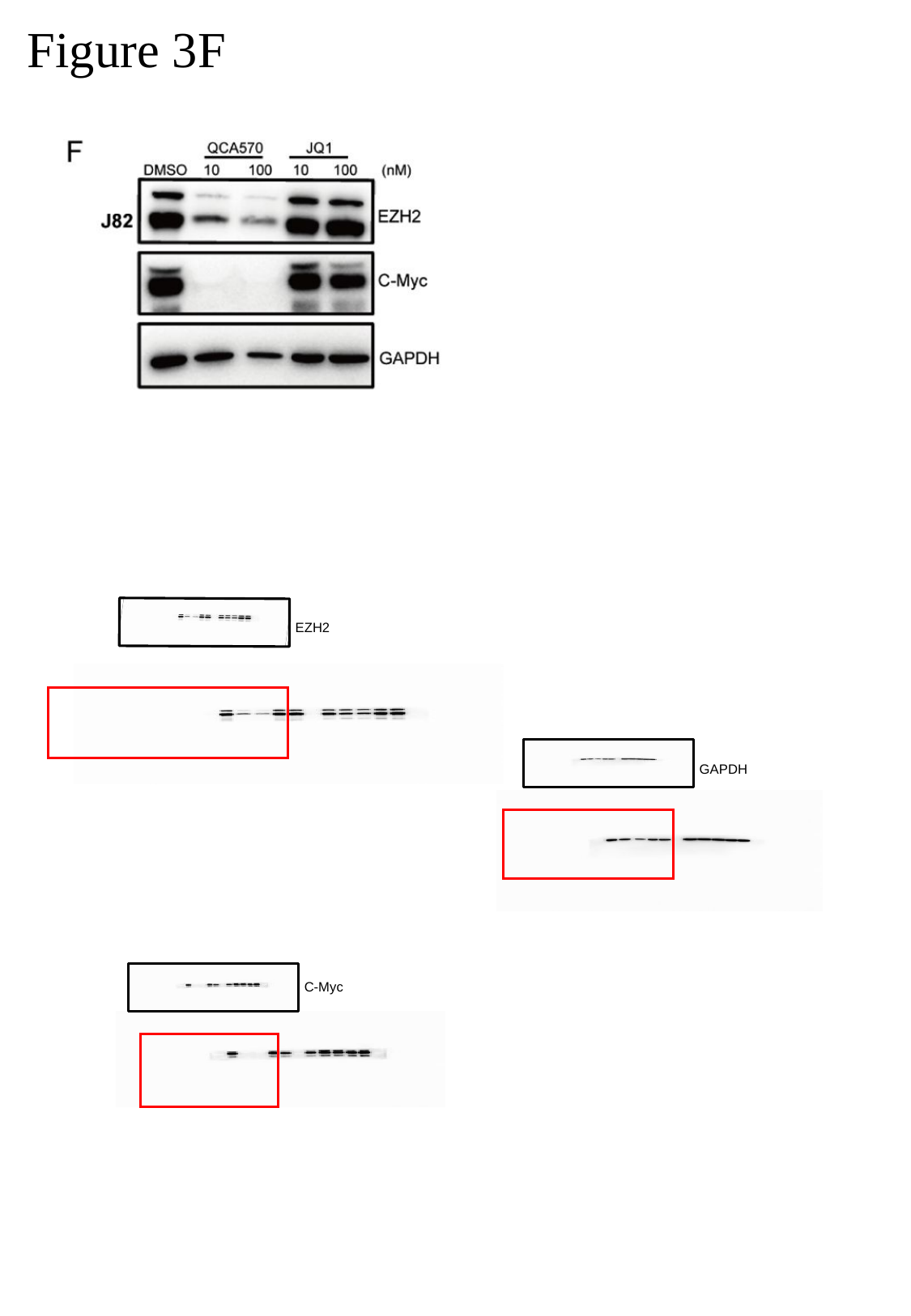

Figure 3F
EZH2
GAPDH
C-Myc

## Slide 3
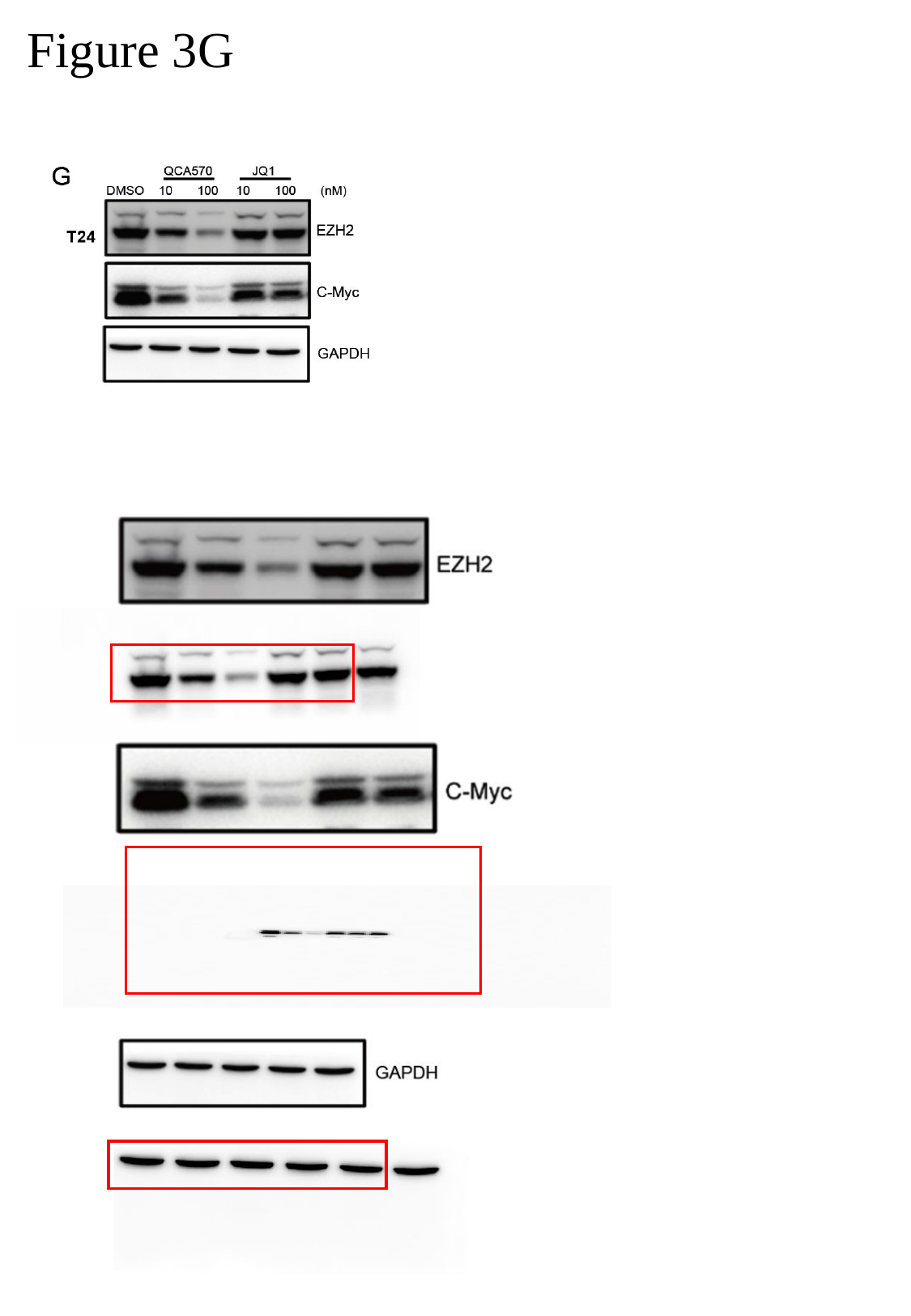

Figure 3G

Supplement: Supplementary file 6 [file DataSheet1.ZIP › Figure 3/Fig3-WB.pptx]

## Slide 1
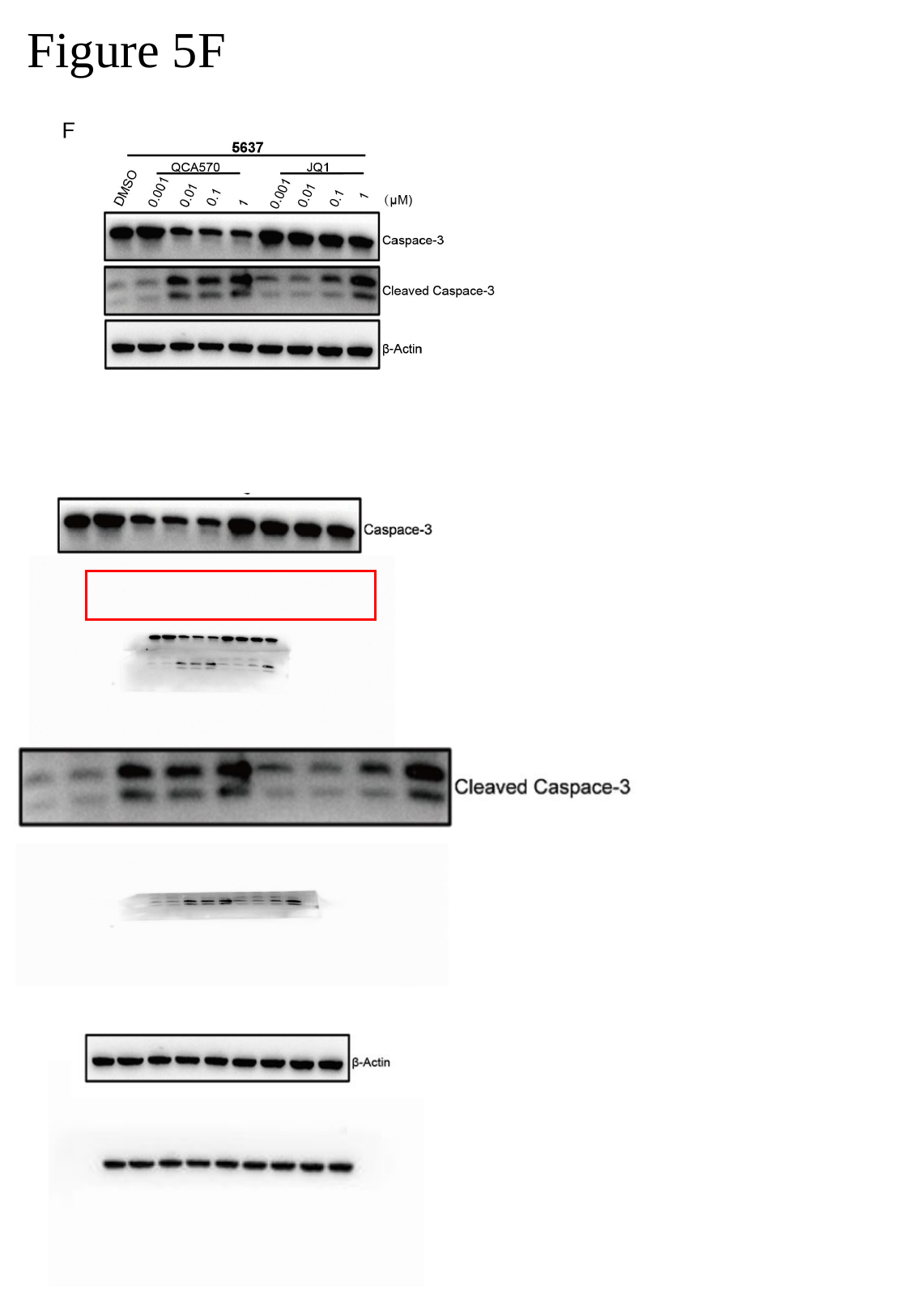

Figure 5F

## Slide 2
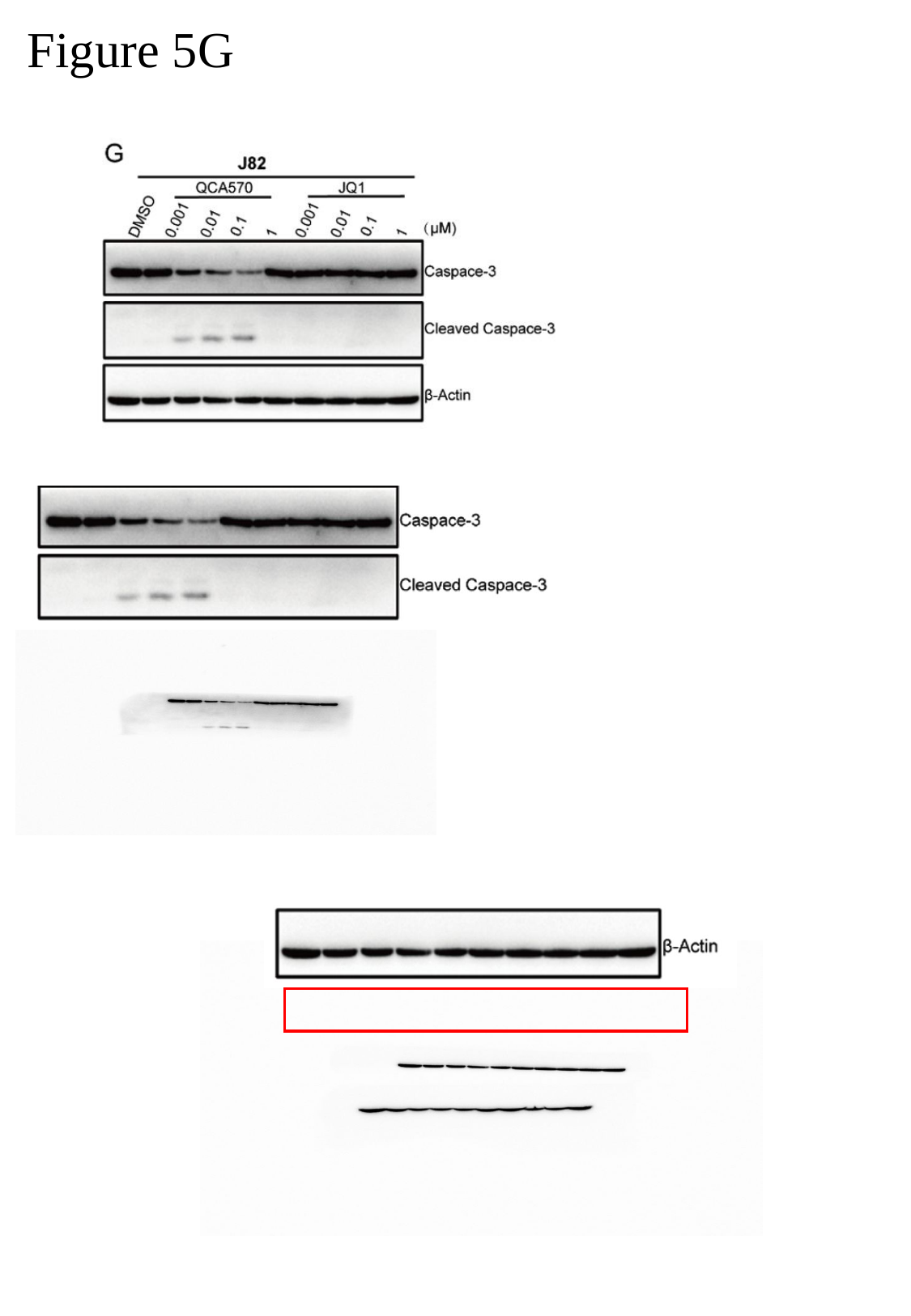

Figure 5G

## Slide 3
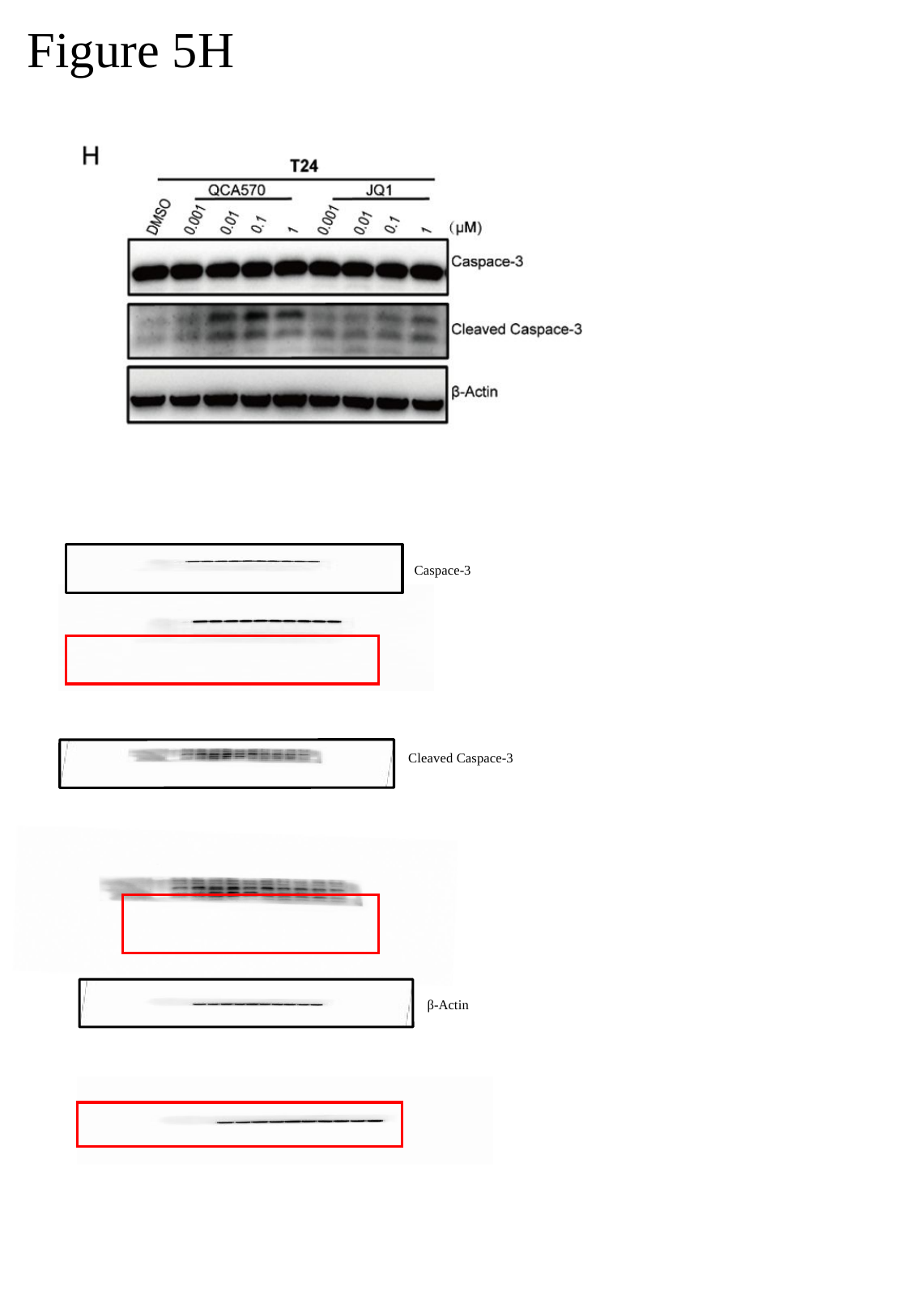

Figure 5H
Caspace-3
Cleaved Caspace-3
β-Actin

Supplement: Supplementary file 7 [file DataSheet10.ZIP › Fig5-F,G,H-WB.pptx]
