# Supplementary material for: Lethal activity of BRD4 PROTAC degrader QCA570 against bladder cancer cells
Source: Front Chem. 2023 Jan 17;11:1121724. doi: 10.3389/fchem.2023.1121724 (PMC9887192; doi:10.3389/fchem.2023.1121724)
Supplement: Supplementary file 6 [file DataSheet1.ZIP › Figure 2/Fig2-WB.pptx]

## Slide 1
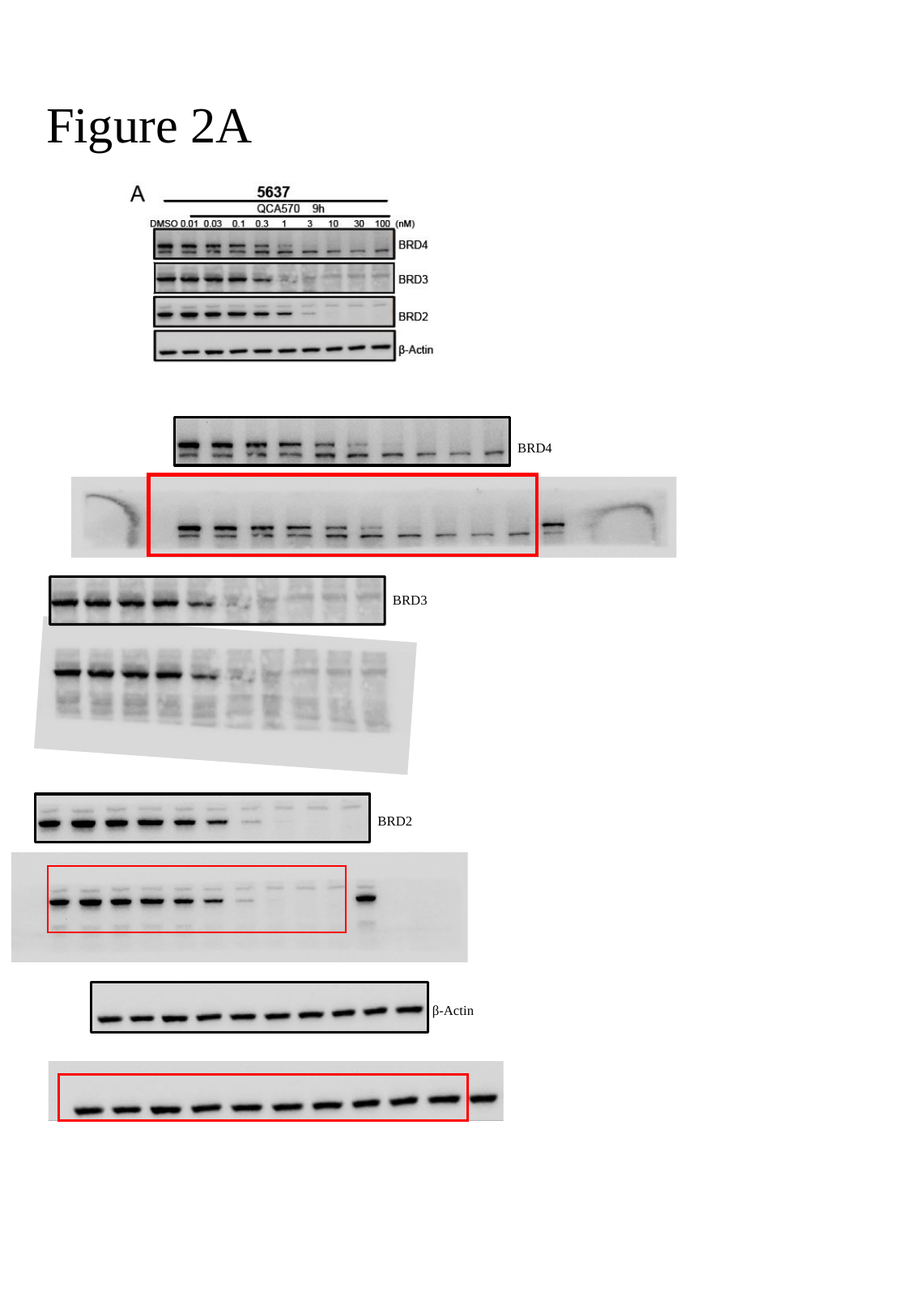

Figure 2A
BRD4
BRD3
BRD2
β-Actin

## Slide 2
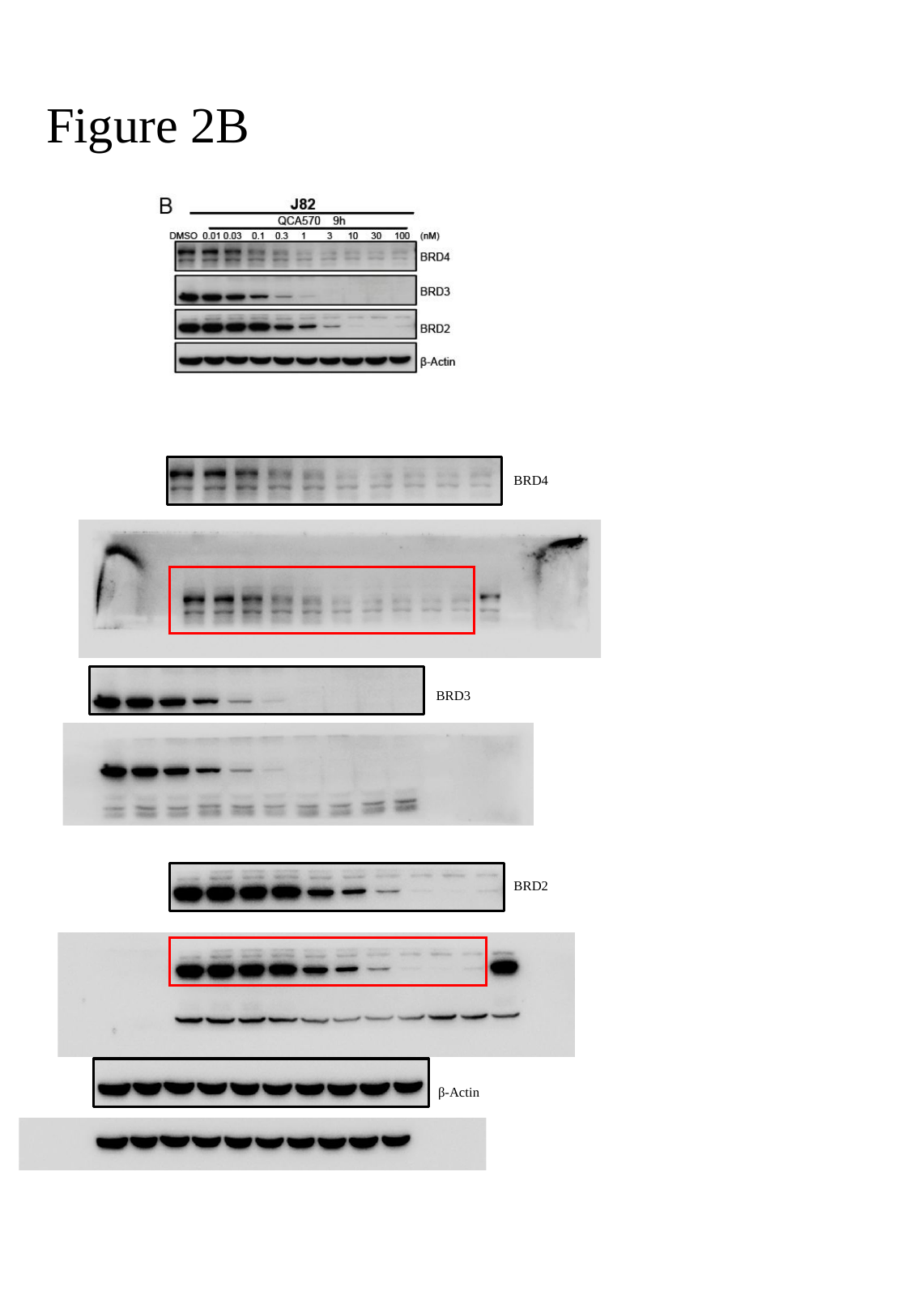

Figure 2B
BRD4
BRD3
BRD2
β-Actin

## Slide 3
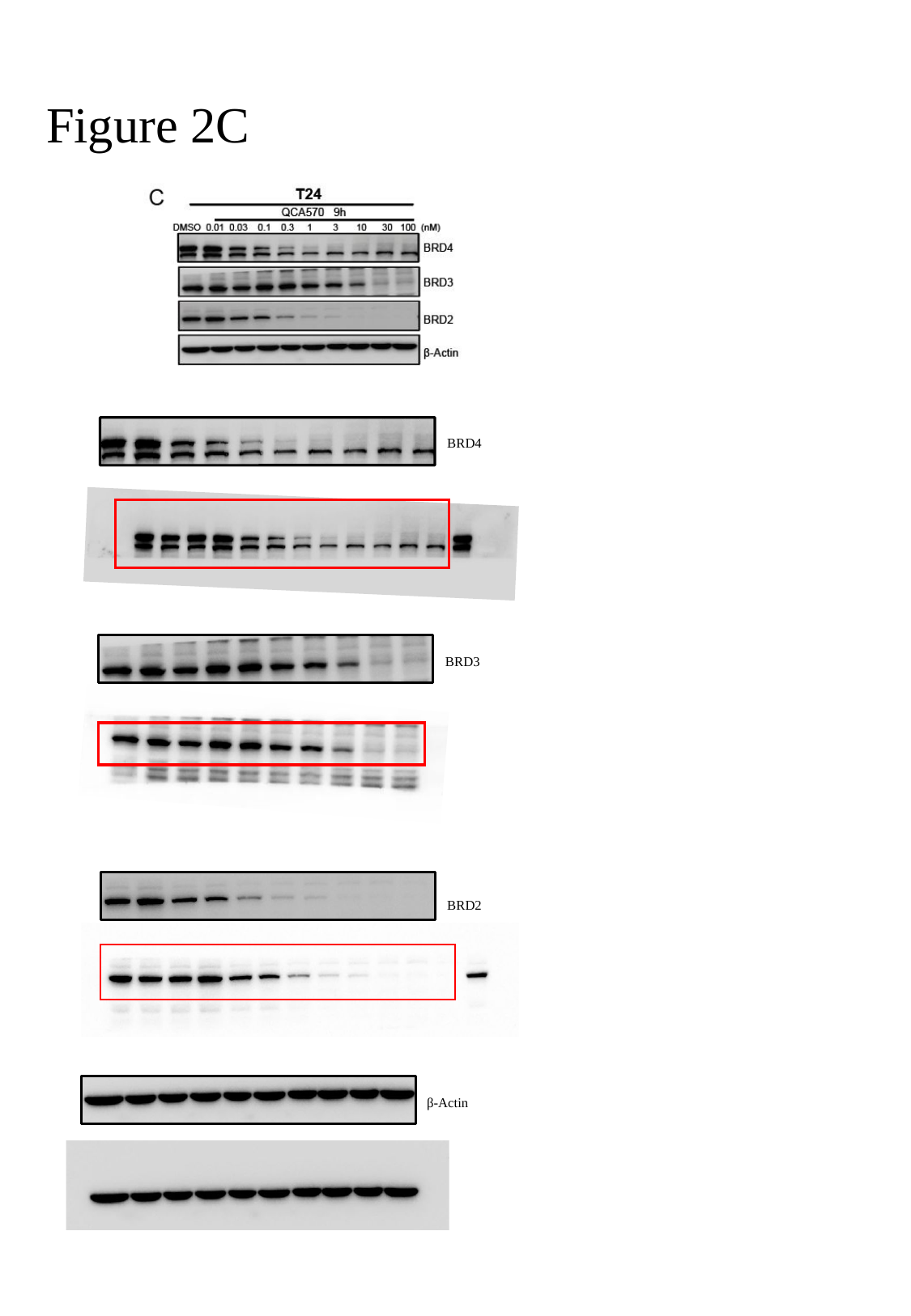

Figure 2C
BRD4
BRD3
BRD2
β-Actin

## Slide 4
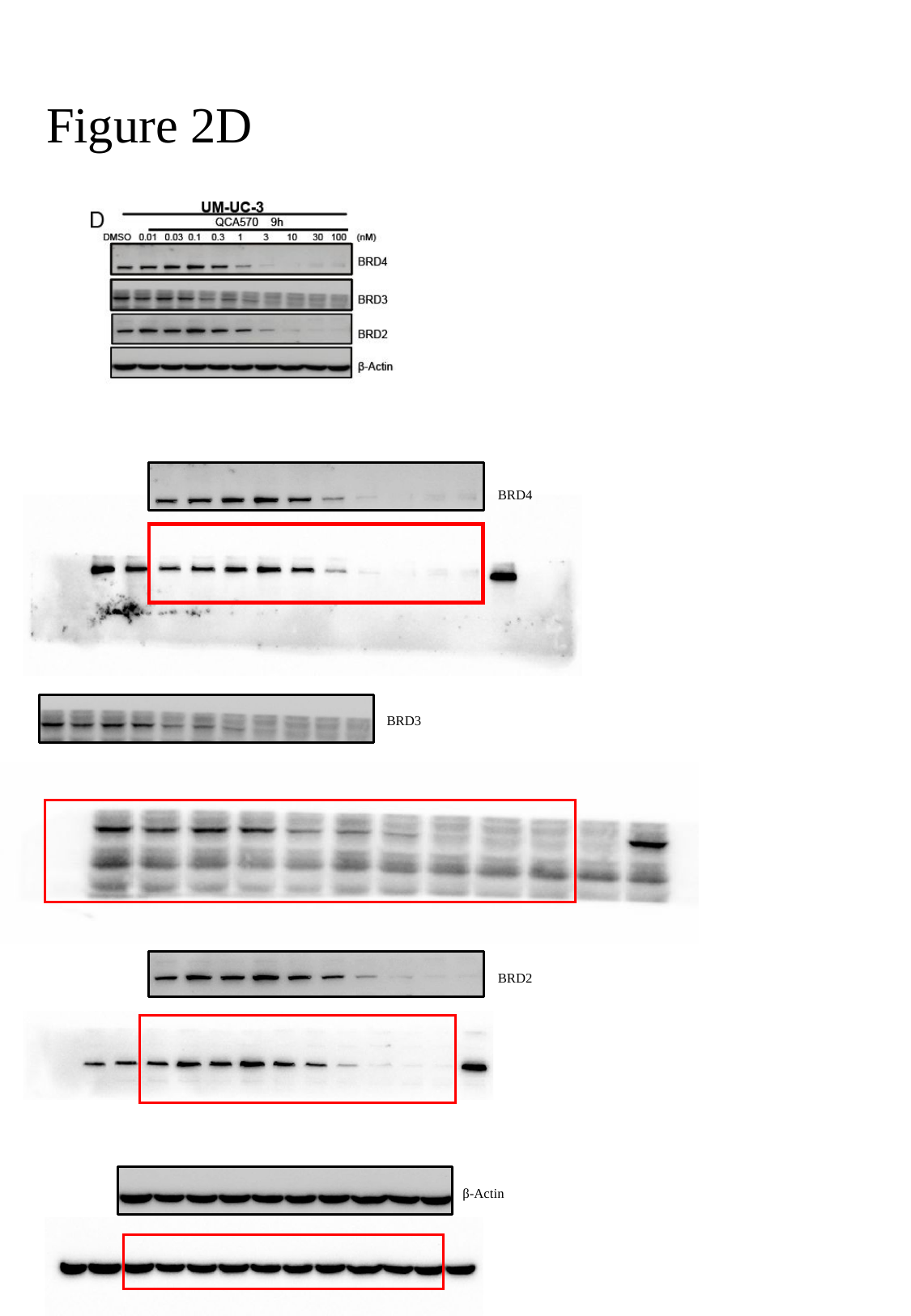

Figure 2D
BRD4
BRD3
BRD2
β-Actin

## Slide 5
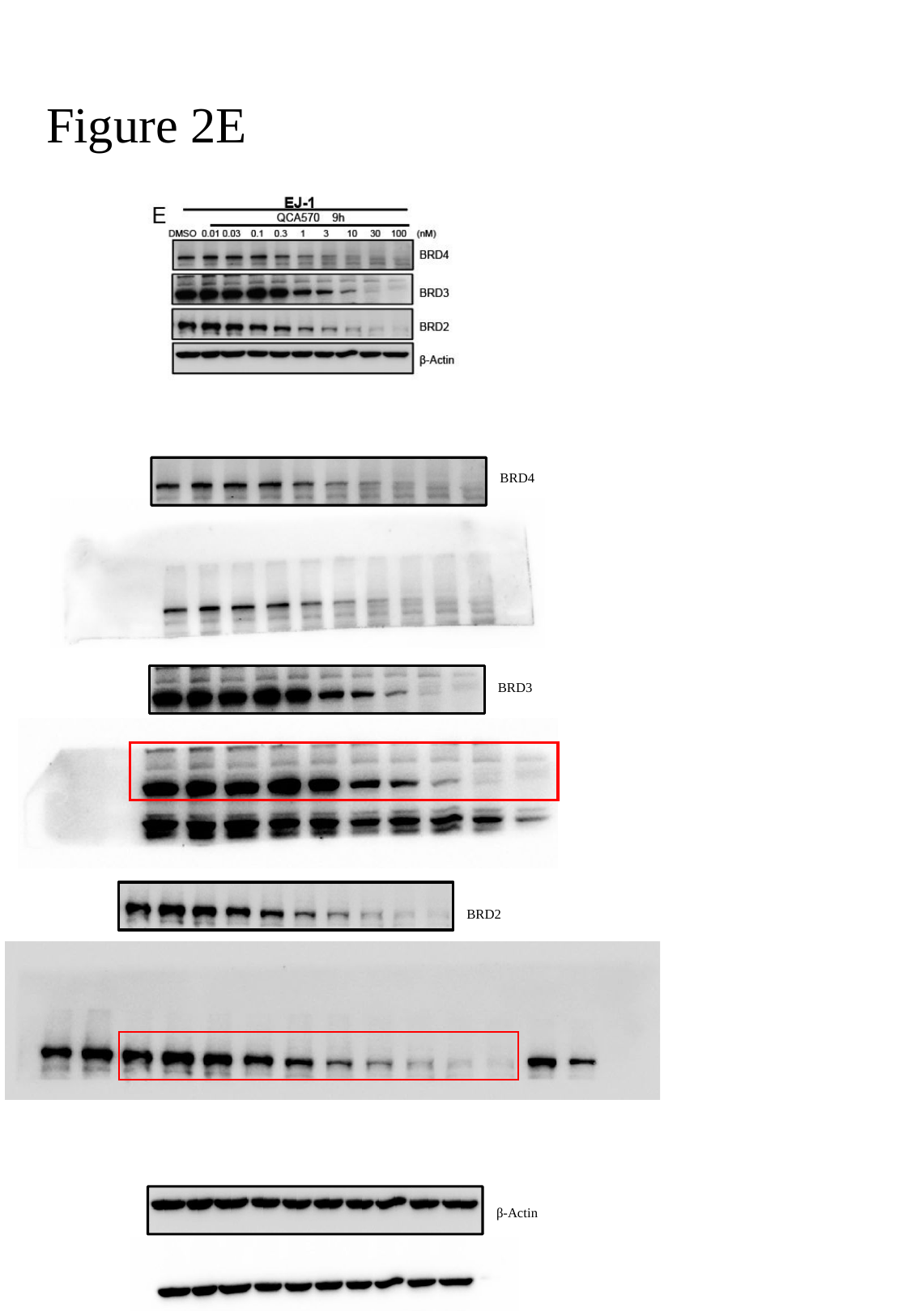

Figure 2E
BRD4
BRD3
BRD2
β-Actin

## Slide 6
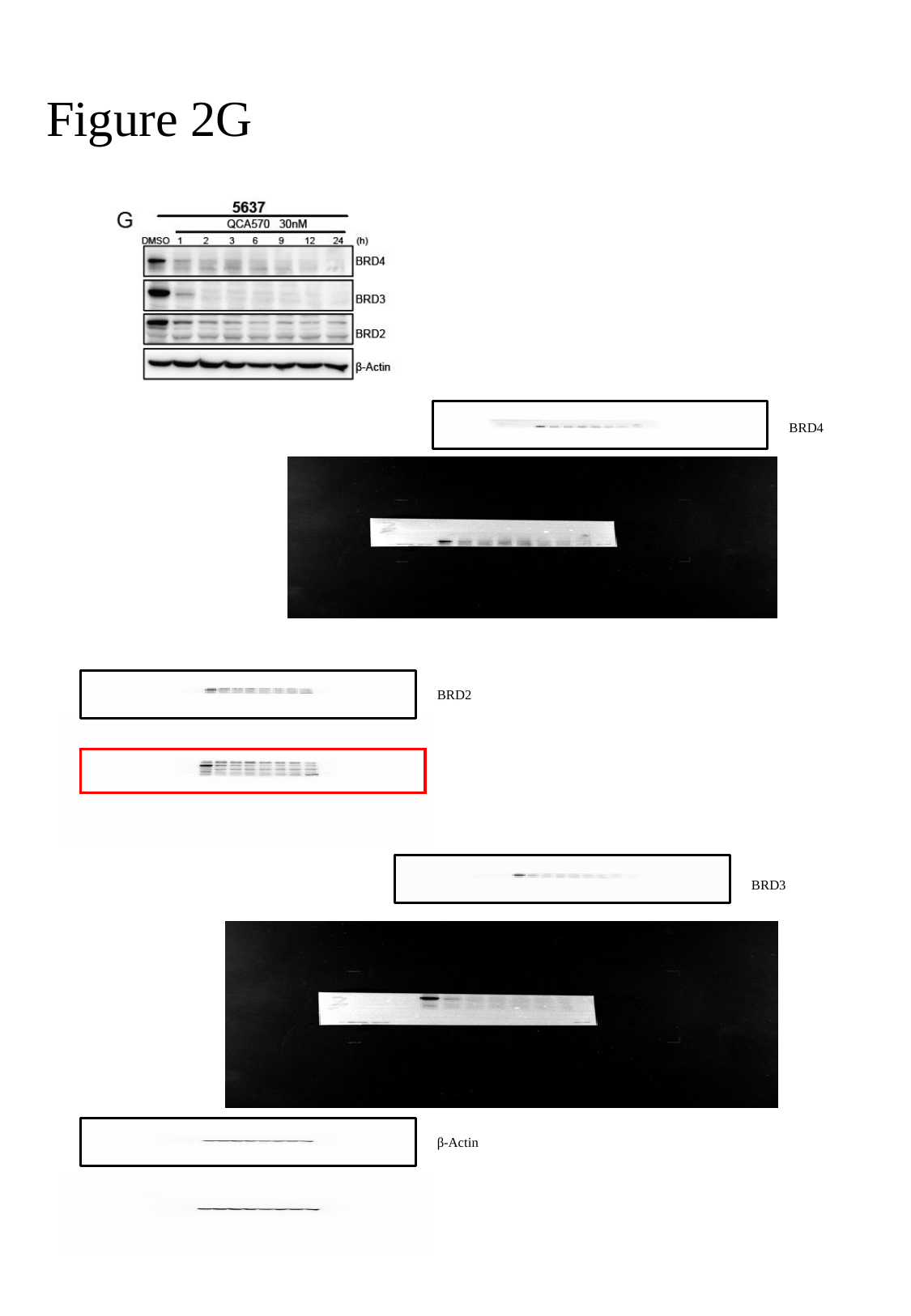

# Figure 2G
BRD4
BRD2
BRD3
β-Actin

## Slide 7
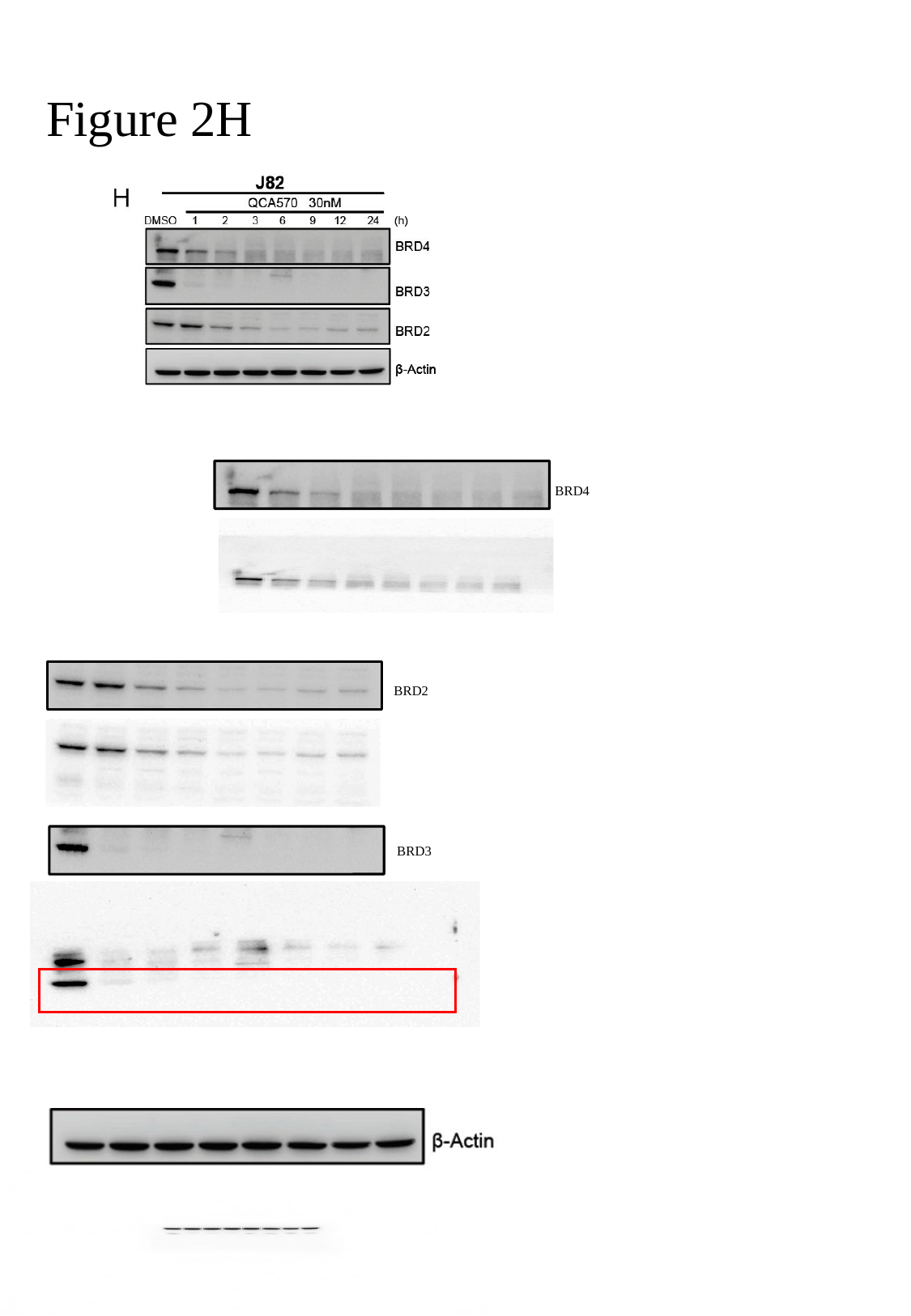

# Figure 2H
BRD4
BRD2
BRD3

## Slide 8
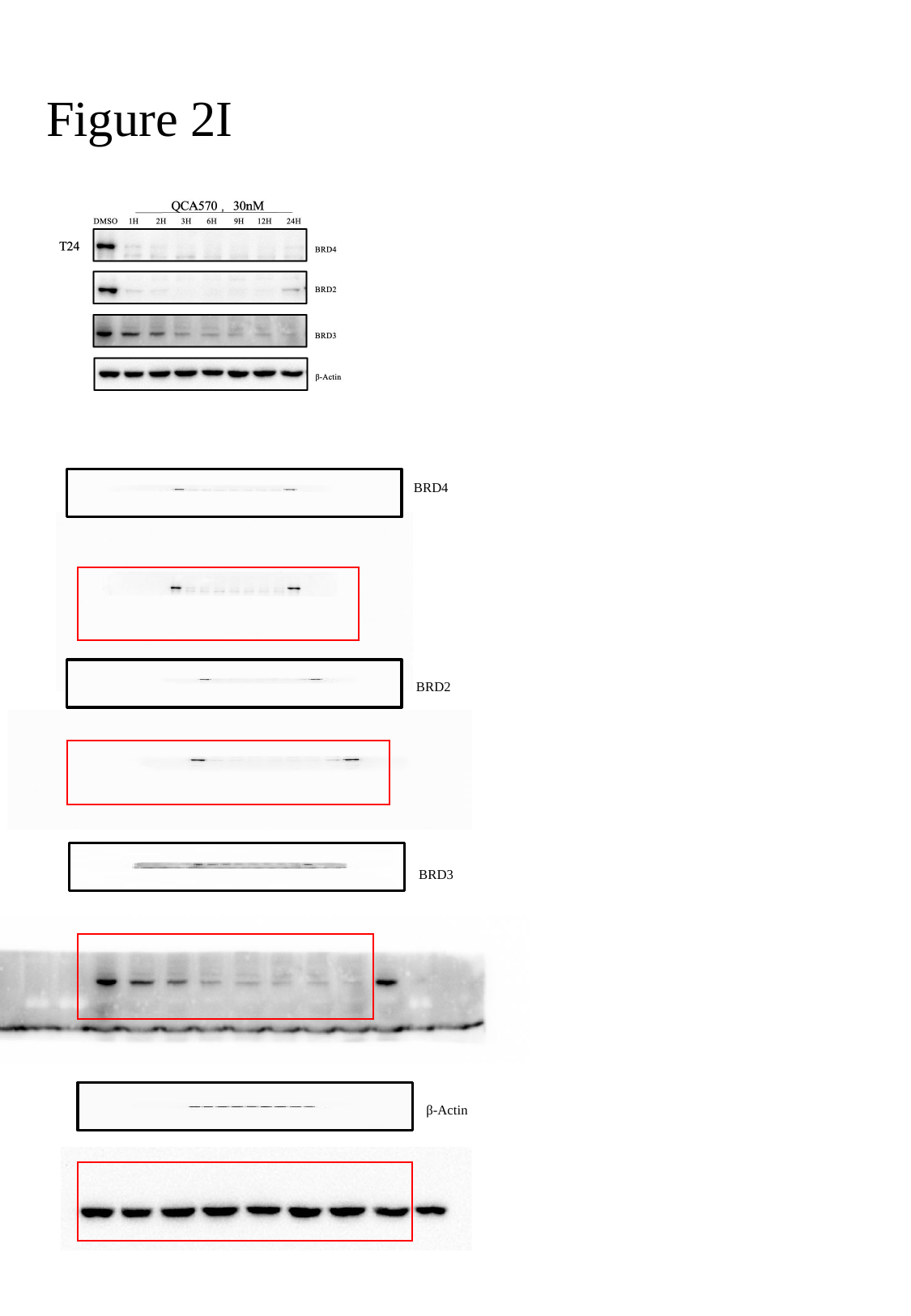

# Figure 2I
BRD4
BRD2
BRD3
β-Actin

## Slide 9
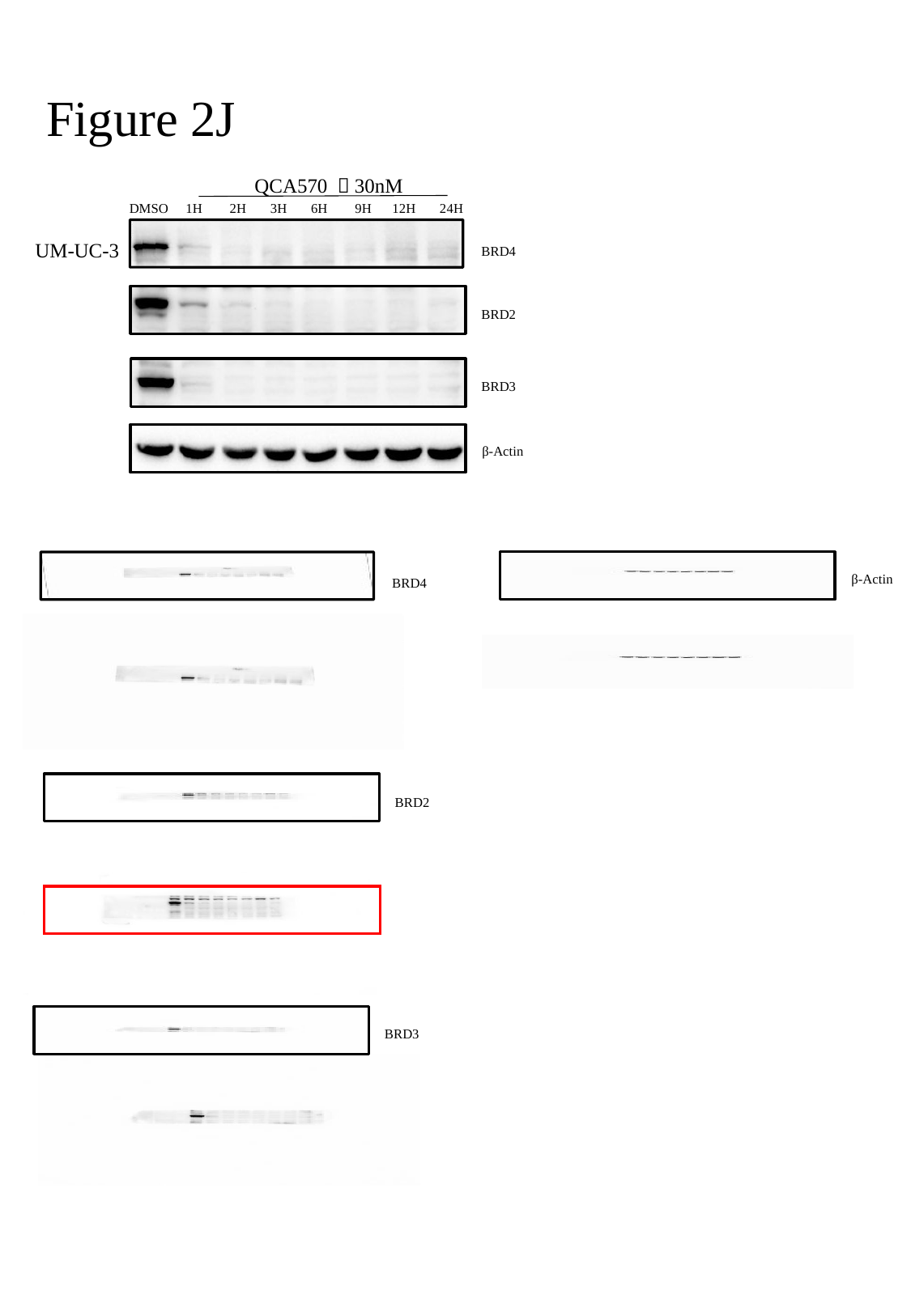

# Figure 2J
QCA570 ，30nM
DMSO 1H 2H 3H 6H 9H 12H 24H
UM-UC-3
BRD4
BRD2
BRD3
β-Actin
BRD4
β-Actin
BRD2
BRD3

## Slide 10
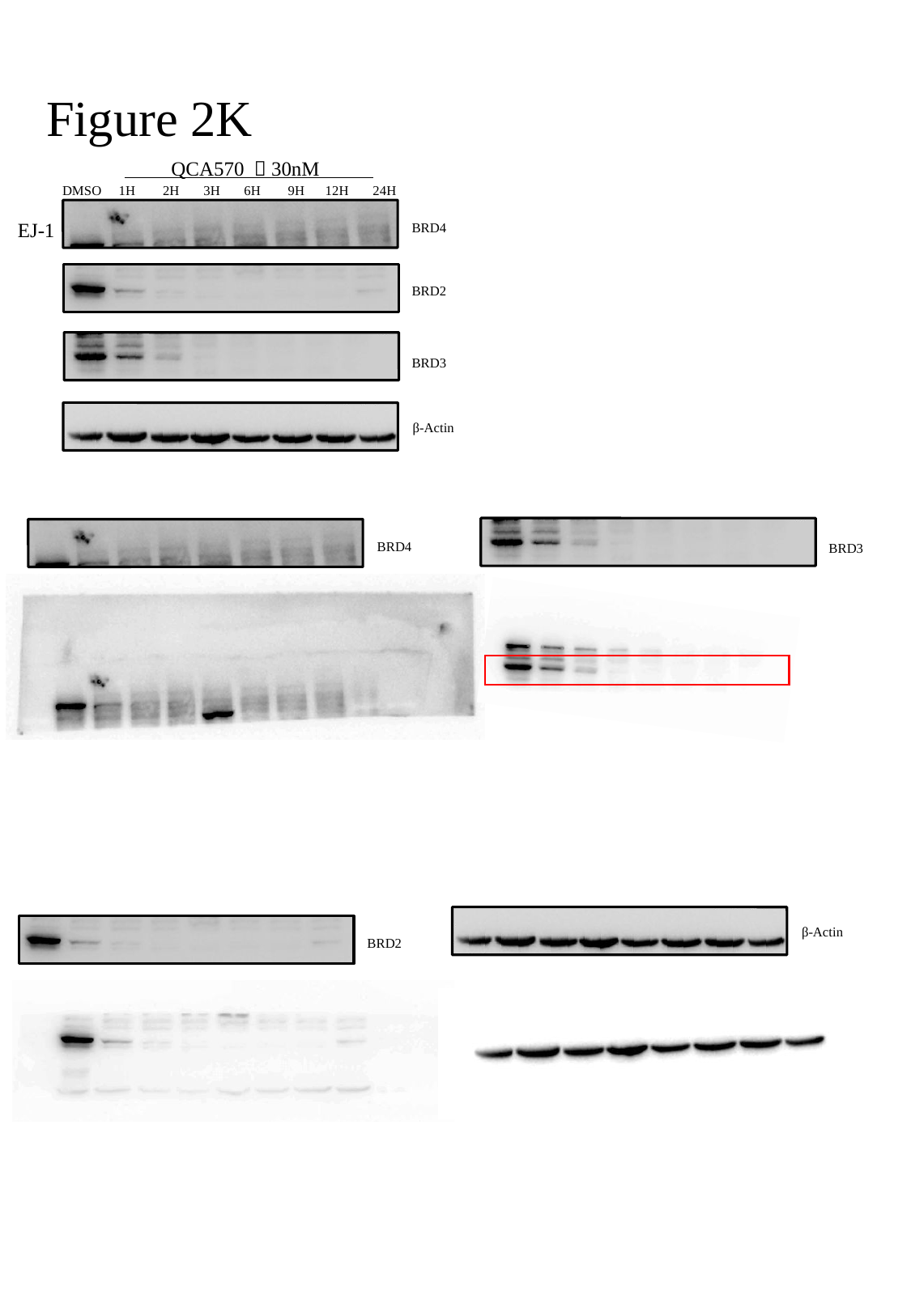

Figure 2K
QCA570 ，30nM
DMSO 1H 2H 3H 6H 9H 12H 24H
EJ-1
BRD4
BRD2
BRD3
β-Actin
BRD4
BRD3
β-Actin
BRD2

## Slide 11
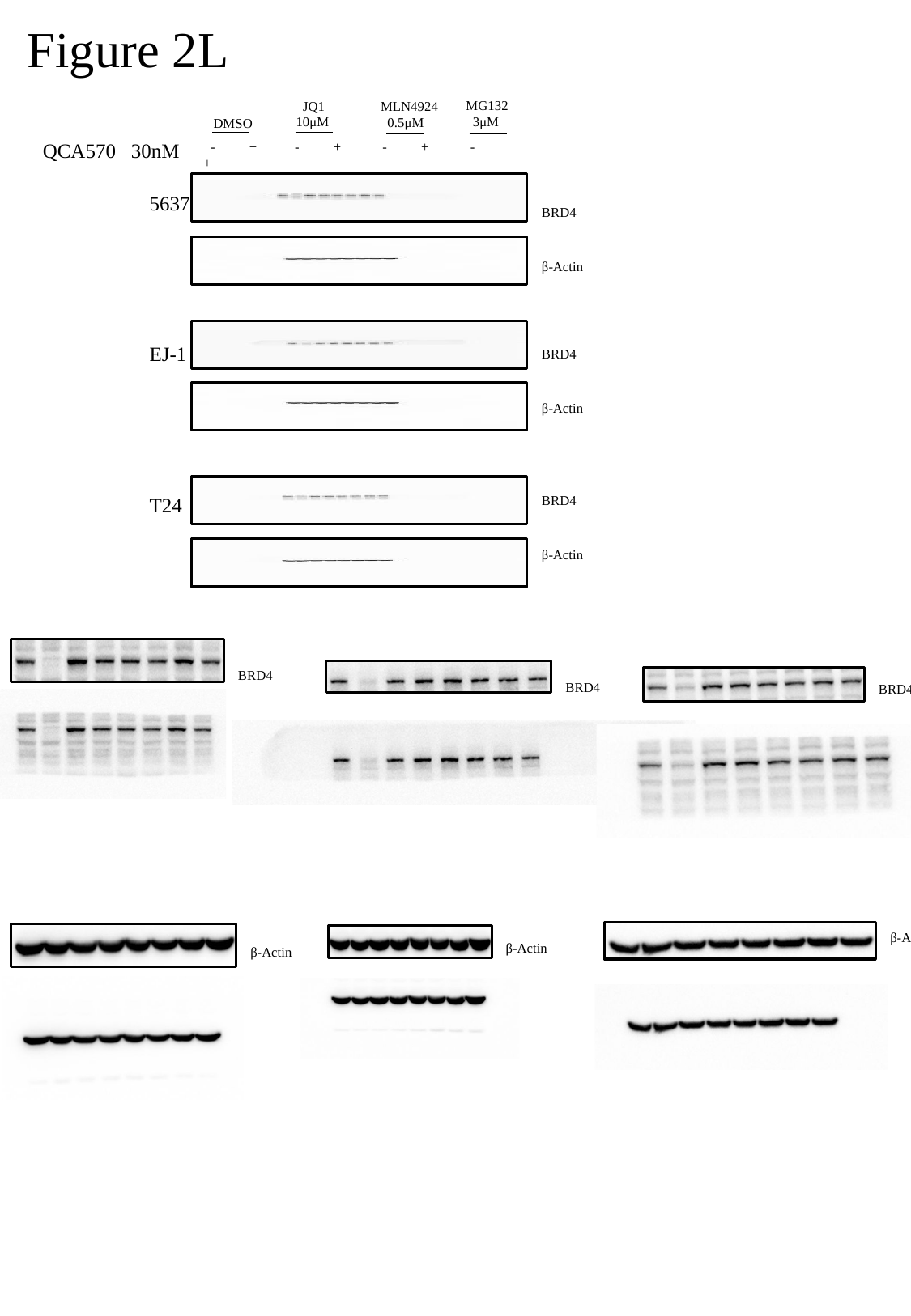

Figure 2L
MG132
 3μM
 JQ1
10μM
MLN4924
 0.5μM
DMSO
QCA570 30nM
 - + - + - + - +
5637
BRD4
β-Actin
EJ-1
BRD4
β-Actin
T24
BRD4
β-Actin
BRD4
BRD4
BRD4
β-Actin
β-Actin
β-Actin
